# High-Throughput Analysis of NF-κB Dynamics in Single Cells Reveals Basal Nuclear Localization of NF-κB and Spontaneous Activation of Oscillations

**DOI:** 10.1371/journal.pone.0090104

**Published:** 2014-03-04

**Authors:** Samuel Zambrano, Marco E. Bianchi, Alessandra Agresti

**Affiliations:** 1 San Raffaele University, Milan, Italy; 2 San Raffaele Scientific Institute, Genetics and Cell Biology Division, Milan, Italy; Baylor College of Medicine, United States of America

## Abstract

NF-κB is a transcription factor that upon activation undergoes cycles of cytoplasmic-to-nuclear and nuclear-to-cytoplasmic transport, giving rise to so called “oscillations”. In turn, oscillations tune the transcriptional output. Since a detailed understanding of oscillations requires a systems biology approach, we developed a method to acquire and analyze large volumes of data on NF-κB dynamics in single cells. We measured the time evolution of the nuclear to total ratio of GFP-p65 in knock-in mouse embryonic fibroblasts using time-lapse imaging. We automatically produced a precise segmentation of nucleus and cytoplasm based on an accurate estimation of the signal and image background. Finally, we defined a set of quantifiers that describe the oscillatory dynamics, which are internally normalized and can be used to compare data recorded by different labs. Using our method, we analyzed NF-κB dynamics in over 2000 cells exposed to different concentrations of TNF- α α. We reproduced known features of the NF-κB system, such as the heterogeneity of the response in the cell population upon stimulation and we confirmed that a fraction of the responding cells does not oscillate. We also unveiled important features: the second and third oscillatory peaks were often comparable to the first one, a basal amount of nuclear NF-κB could be detected in unstimulated cells, and at any time a small fraction of unstimulated cells showed spontaneous random activation of the NF-κB system. Our work lays the ground for systematic, high-throughput, and unbiased analysis of the dynamics of transcription factors that can shuttle between the nucleus and other cell compartments.

## Introduction

The tight control of transcription factors activity is mandatory to warrant an adequate cell response to environmental cues. Several transcription factors are located in the cytosol or associated to membranes, and are activated by specific signaling pathways to enter the nucleus where they activate the transcription of specific genes. Most analyses of these activation processes are done at cell population level, for example by immunoblotting nuclear and cytoplasmic fractions. However, single-cell analysis gives a wealth of additional information: for example, cells may not respond synchronously to the signal, and some cells may not respond at all. In addition, some signaling pathways give rise to oscillating behaviors of proteins that are imported and exported from the nucleus several times: examples are NF-κB, p53 and ERK in mammalian cells [1], [2], [3], [4], and Ace1p and Msn2 in yeast [5], [6]. Such oscillating behavior might encode biological information itself, not unlike calcium oscillations [7].

The members of the NF-κB family of transcription factors (homo/hetero dimers of p65, p50, p52, cRel and RelB) coordinately control hundreds of genes [8] that play a pivotal role in multiple steps of inflammation, from microbial killing to endothelial activation. Upon inflammatory stimuli (e.g. with TNF-α or LPS), IκB inhibitor proteins that constrain NF-κB in the cytoplasm of resting cells are degraded, and NF-κB relocates into the nucleus where it drives the expression of many genes, including those encoding the IκB inhibitors [2]. This gives rise to a negative feedback loop. The intrinsic time lag between IκB gene activation and accumulation of the proteins causes the system to display oscillatory dynamics that has been observed at single-cell level [1], [9], [10]. Different combinations of stimuli and drugs affect NF-κB dynamics and lead to different transcriptional responses [9], [10], [11].

However, we are still far from having a complete picture of these complex dynamics, mainly because the variety of quantifiers and units that have been used to describe the system made the datasets non-comparable. Similarly, the description of the observed dynamics has remained somehow vague, and the observed “oscillating” or “responding” behavior lacks a unified cell type-independent definition.

To extract meaningful information on cell dynamics, hundreds of cells must be analyzed in a single experiment, requiring automated processing of time-lapse microscopic imaging that keeps track of moving cells over long times. The relative amount of nuclear and total protein must be expressed with univocal metrics, and the time evolution of measurements must be captured with simple indexes.

Our work aims at filling these technical gaps by providing descriptors and quantifiers for a univocal and cell type-independent characterization of the dynamics upon different stimulations. The effect of different stimuli on NF-κB dynamics can then be quantitatively evaluated without bias in experiments performed with heterogeneous set-ups. Our procedure can be applied to cells that are sparse enough to allow a precise segmentation of their cytoplasm. Our segmentation is based on an accurate estimation of the background in the vicinity of the cell, without pre-assuming any particular cell shape, in contrast with the method recently proposed by Di et al. [12] where the shape of the cytoplasm of clustered cells needs to be approximated.

This approach allowed us to better describe the oscillatory dynamics of NF-κB, to uncover some novel features and to have a deeper insight on others that were previously overlooked.

### Rationale

To obtain a unified definition of the NF-κB oscillatory response, we have developed a method and its software implementation to rigorously describe the dynamics of fluorescently labeled NF-κB molecules in hundreds of cells using time-lapse microscopy. To represent GFP–p65 cellular distribution we use the ***Nuclear to Total ratio*** of the fluorescent signal that we call ***NT***. This *quantifier* takes into account the overall amount of NF-κB in each cell and the fraction that relocates into the nucleus as a function of time. *NT* can be considered a cell type-independent and internally normalized quantifier: it varies between 0 (for a cell with no nuclear NF-κB) and 1 (for a cell where all NF-κB is nuclear). Moreover, the procedure for *NT* calculation corrects for most of the experimental distortions that might happen throughout acquisition. Our method was tested with mouse embryonic fibroblasts (MEFs) from a GFP-p65 knock-in mouse [9], [13]. MEFs expressing GFP-p65 at physiological levels are very dim and their fluorescence is barely detectable using a standard wide-field illumination. When fluorescence intensity is extremely low and close to the limit of detection as in these cells, a rigorous evaluation of the image background and signal intensities is crucial for *NT* quantification. For this reason, our software includes a procedure for a careful evaluation of the background intensity in the proximity of each cell.

Our analysis allowed us to standardize the evaluation of known dynamics and to report on new features that to our knowledge went unnoticed.

### This Paper Is Organized As Follows

***Section I*** provides a description of the method. ***Section I.A*** describes how we compute the ***background***, perform cell segmentation and track hundreds of cells. ***Section I.B*** describes our method for a quantitative analysis of the dynamics. We propose to use ***NT*** (*Nuclear to Total ratio*) as a cell type-independent and internally normalized quantifier of NF-κB dynamics.

***Section II*** describes the results obtained applying our method to GFP-p65 knock-in cells. In ***Section II.A*** the performance of the method is discussed, and we present high-throughput data showing that unstimulated cells present ***non-zero basal levels*** of nuclear NF-κB. ***Section II.B*** identifies univocal **descriptors** for NF-κB dynamics. With this procedure, we recover the dose-dependent response of cells upon TNF-α stimulation. In ***Section II.C*** the precise definition of descriptors of NF-κB activity allows us to conclude that unstimulated cells also present ***spontaneous NF-***κ***B activity***. In ***Section II.D*** we ***characterize the dynamics*** for cells upon different doses of TNF-α using our descriptors. Finally we draw the main conclusions of this work.

### Section I: Description Of The Quantification Method

#### I.A Cell Segmentation, Tracking and Background Estimation

**Figure 1:**
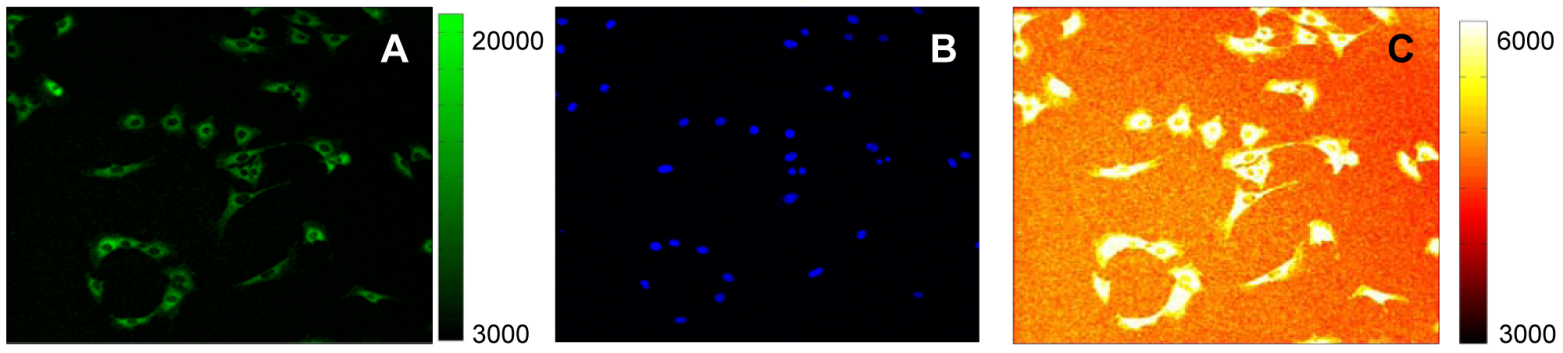
Images used for quantification of the GFP-p65 relocation dynamics. (**A**) Low magnification (20×) image taken at time 0 in the GFP channel showing a representative field from a time-lapse acquisition experiment. GFP-p65 is mostly localized in the cytoplasm of unstimulated cells. Color-map and scale are shown on the right. (**B**) HOE channel acquisition of the same field as in A. Nuclei counterstained with the nuclear dye Hoechst 33342 appear as bright blue spots. (**C**) The GFP image as in A is shown using an adjusted color-map (on the right) The scale is extremely compressed for high GFP intensities (white for values ranging from 6.000 to 20.000) but expanded for values that approximate the background (orange). With these settings, variations in the background intensities can be appreciated; it is reasonable to presume that spatial variation exists for the GFP-p65 signal as well. The background is roughly of one order of magnitude less, but it is high enough to be taken into account, most importantly for the quantification of very low signals as for the nuclear non-zero basal level in unstimulated cells.

As a first step, we established a procedure to extract reliable quantitative information on the localization of p65. In order to identify the nucleus, cells were stained with 50 ng/ml of the vital dye Hoechst 33342, less than half the concentration used typically for living cells [14], [15] (see Materials and Methods). We used a less energetic 405 nm laser than the near or far UV lasers. We also checked for phototoxicity [16] by recording for 15 hours Hoechst-stained (Video S1 and S2) and non-stained cells (Video S3 and S4), finding equally low death rates (close to 5%). We do not find other evidence of phototoxicity, such as variation of mitosis duration or segregation defects [17]. Images taken at each time-point (Figure 1B, HOE channel) also allow the tracking of each cell during the time-lapse acquisition.

At each time-point a GFP image is also collected to quantify the GFP-tagged p65 (GFP channel, **Figure 1A**). Robust and reliable quantification of the dynamics requires to capture fluorescence coming from the whole cell. We achieved this goal by acquiring images with a z-slice thickness of about 10 µm, comparable to the total cell thickness **(**Materials and Methods). This technical detail avoids DNA damage, NF-κB activation and signal bleaching due to excessive photo-exposure.

Background is then estimated: this step is of paramount importance when the signal intensity is low. In fact, the use of a sensitive color-map scale for the GFP image reveals variations in the background intensity (**Figure 1C**, compare the top-right low-intensity corner with the bottom-left higher intensity corner).

**Figure 2:**
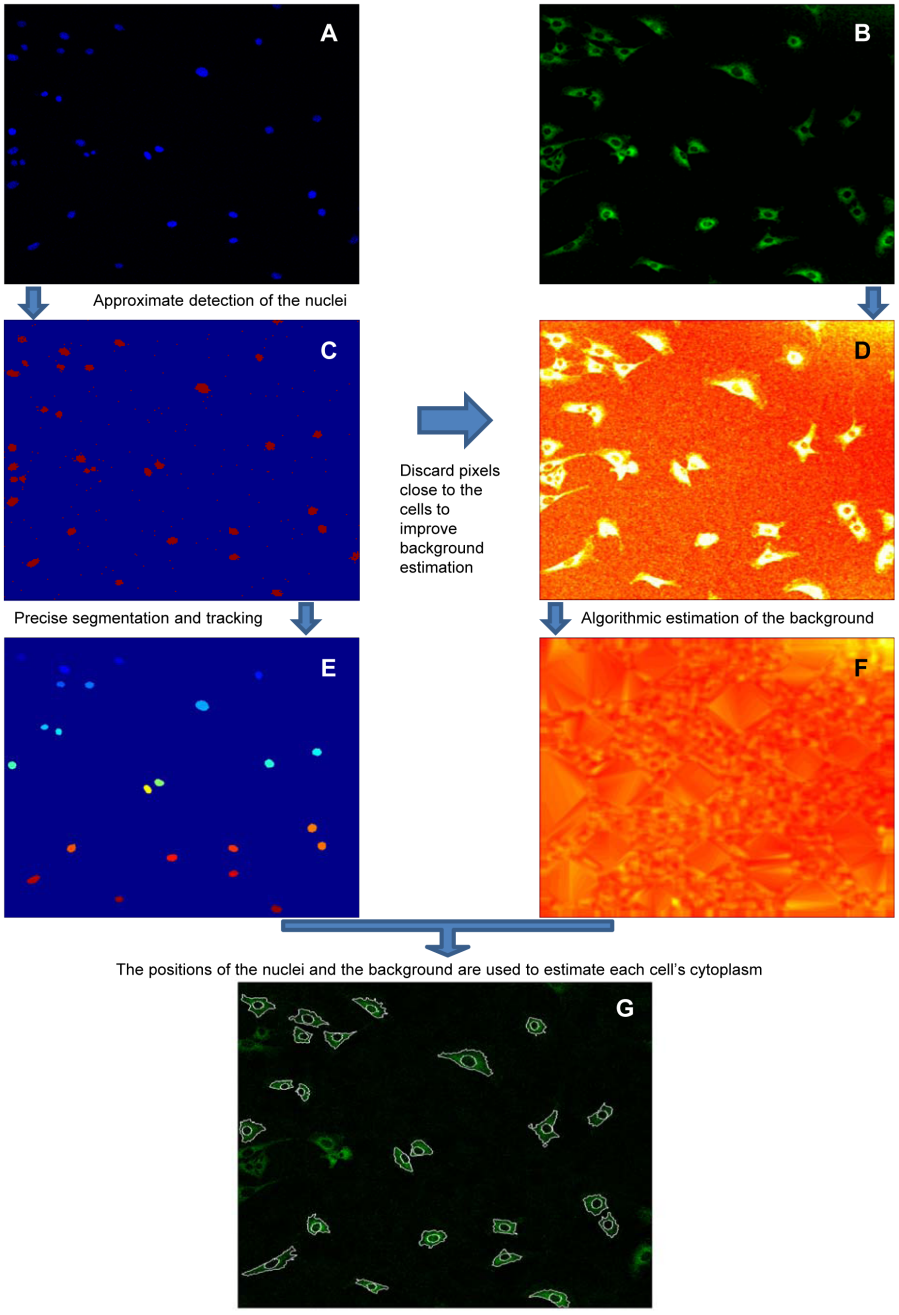
Workflow of the automated method for the quantification of nuclear-to-cytoplasmic dynamics of GFP-p65. For each frame we generate two images, a HOE image of the stained nuclei (**A**) and a GFP image of GFP-p65 (**B**). (**C**) The HOE image shown in **A** (pseudocolors) is divided in tiles and by a K-means algorithm we determine a threshold used for an approximate detection of the nuclei. (**D**) The approximate position of the cells – derived from the approximate nuclei position – is taken into account for the estimation of the background of the images, which is spatially inhomogeneous. (**E**) The segmentation of the nuclei is refined by computing the average brightness of each nucleus that is used to determine a nucleus-specific threshold (the colorcode is based on the incremental identification of nuclei). (**F**) An algorithm is used to reconstruct the background for the full image. Of note, the background clearly varies in space. (**G**) Using the local background estimation we identify the boundaries of each cell. The nuclear to total ratio (*NT)* of the GFP-p65 protein is then calculated using the segmentation of nucleus and cytoplasm of each cell as described in the Results.

The whole quantification procedure is summarized in **Figure 2** in which panels **A** and **B** are the HOE and GFP channel images, respectively. Here we give a brief summary; the software is described in further detail in **Document S1.** The code is available upon request.

#### Nuclei identification

First, the HOE image (1024×1024 pixels) is divided in identical square tiles (32×32 pixels) and we use a K-means clustering of the intensities to give an approximate value of the HOE nuclear fluorescence intensities, allowing the approximate detection of all the nuclei in the image (**Figure 2C**). A more precise segmentation of nuclei is obtained by using a threshold computed from the average brightness of the HOE signal in the vicinity of each nucleus (**Figure 2E**). This is useful because typically the nuclei are unevenly stained (see **Figure 1B** and **2A**). Each nucleus is then tracked through the time-lapse images by connecting it to the closest one in the previous frame (procedure details in **Document S1**). If the geometry of the nucleus has varied a lot – indicative of a cell undergoing mitosis or apoptosis – the cell is discarded (parameters can be adjusted by the user).

#### Whole cell segmentation through background estimation in the GFP channel

Whole cell boundaries are outlined by the pixels with intensity higher than the background, a key point in our methodology. The background is calculated by an algorithm [18] that first divides the GFP image in tiles as described before for the HOE image. Then, a clustering based on statistical properties of the remaining tiles detects those belonging to the background. Accuracy is further enhanced by discarding those tiles that contain the approximate positions of the cells (using the information from **Figure 2C**). Finally, the gaps corresponding to discarded tiles are filled by interpolation. With this approach we are able to estimate the background in the whole image, as shown in **Figure 2F**.

To segment the cell boundaries we identify the pixels belonging to the cytoplasm as those that have intensities higher than the background (Details in **Document S1**). The result of our segmentation procedure is shown in **Figure 2G**.

Note that in contrast to other methods [12] we do not approximate the cell shape by pre-assuming the geometries of the cytoplasms: our definition of the cytoplasm comes from a precise estimation of the local background. This has a drawback, though: when two or more cells touch each other, the segmentation procedure fails and cells are discarded. Implementations based on watershed algorithm as a tool for segmentation can help but would introduce necessarily a bias. The method described here is simple and could in principle be applied to any experimental dataset.

#### I.B Selection of the Quantifier

In the previous section, we identified the regions containing the fluorescence coming from each nucleus and the whole cell. We now propose the Nuclear to Total ratio (NT) as the most reliable quantifier for p65/NF-κB dynamics.

A time-lapse microscopy experiment records a series of images that represents the fluorescence intensity at different points for certain times. This can be represented mathematically as *I(p,t)*, where *p* is the (two-dimensional) coordinate of the pixel and *t* stands for time. This intensity will be the sum of different contributions, which can be written as:
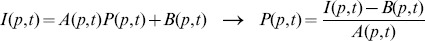
(1)where *P(p,t)* is the amount of protein in the pixel *p*, *A(p,t)* is the amplification coefficient between the protein brightness and the amount of protein, and *B(p,t)* corresponds to the background. Images taken in the GFP channel (**Figure 1C**) show variations in background intensity (compare the top-right low-intensity corner with the bottom-left higher intensity corner). Therefore, the assumption that the amplification coefficient *A(p,t)* and the background *B(p,t)* may vary across the image and in time is fully justified. This might be due to optical distortions, variations in the efficiency of the light detectors or uneven illumination e.g. fluctuations of the laser source within the timescale of the experiment. This would necessarily introduce an error in signal quantification. Moreover, the cells that we are tracking might change their position in time between regions with different values of *A(p,t)*. Our method corrects for this bias.

For this reason, we have to adequately select our quantifier. Note that the total intensity of the nucleus minus the background will not be an adequate quantifier. It will not be proportional to the total amount of the protein in the nucleus:
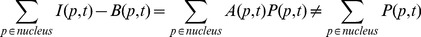
(2)


In other words, in this situation this quantifier would be affected by the distortions mentioned above and thus it is not a good measure of NF-κB relocation dynamics.

From these considerations we have decided to use a quantifier that can overcome these distortions, a quantifier that we refer to in what follows as *NT*:
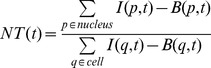
(3)


*NT* is a good approximation of the nuclear to total ratio of the amount of protein, since:
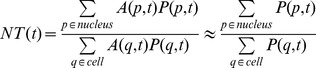
(4)


The approximation to the ratio provided by equation (3) will be valid if *A(p,t)* can be considered constant within the area of the cell, which is a reasonable assumption for our images. As described in the previous section, we have a precise identification of the pixels belonging to each cell and to each cell's nucleus together with a precise calculation of the background, so we can use *I(p,t)* and *B(p,t)* and compute *NT(t)* for each cell as described by equation (3). Although the whole procedure is computationally challenging, the reward is high: this measure will be free of the distortions inherent to time-lapse microscopy experiments.

**Figure 3:**
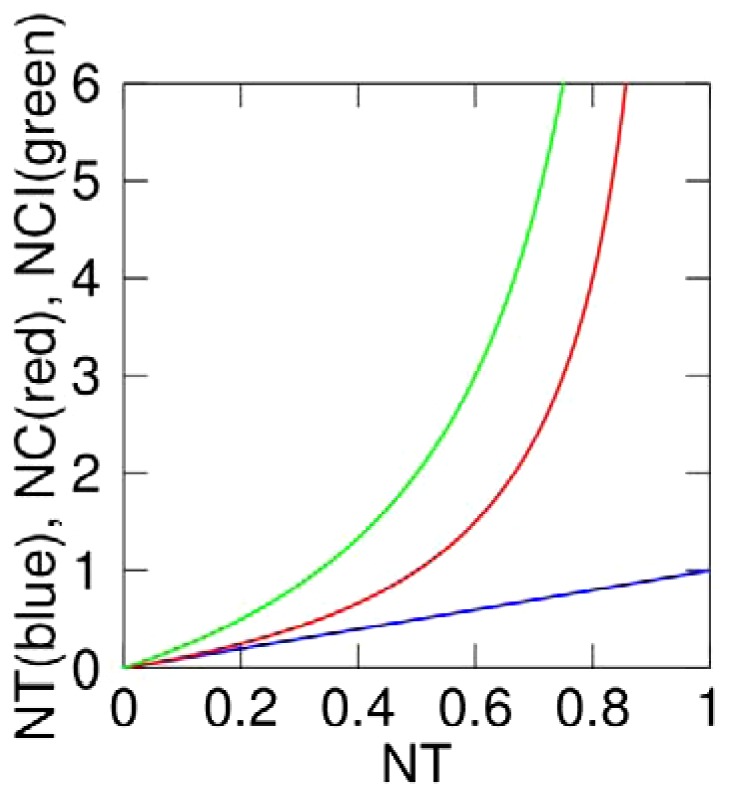
Non-linear relationship between Nuclear to Total Ratio and Nuclear to Cytoplasmatic Ratios. Plot of the values of the Nuclear to Cytoplasmic ratio, *NC* (red), and Nuclear to Cytoplasmic mean Intensity ratio of NF-κB, *NCI* (green), which would be obtained for all the possible values of the Nuclear to Total ratio of NF-κB, *NT* (also plotted, in blue). The relationship between the magnitudes is clearly nonlinear, note that *NC* and *NCI* diverge as *NT* gets close to 1. Details on how these magnitudes can be related are given in **Document S2**.

It is possible to relate our *NT* quantifier with the Nuclear to Cytoplasmic ratio of NF-κB that has been used as a quantifier of the dynamics in a number of works [1], [9], [11], [12], [19]. This relation can be found either as a ratio of the “nuclear to cytoplasmic *total* intensities”, quantifier that we denote *NC*, or as the “nuclear to cytoplasmic *average* intensities”, that we denote *NCI* (details are given in **Document S2**). Thus, if calculated properly, these quantifiers are as robust to experimental distortions as *NT*. However the relation between both *NC* and *NCI* with *NT* is strongly nonlinear, as shown in **Figure 3**. Furthermore, when *all* NF-κB is in the nucleus (so *NT* = 1) the *NC* and *NCI* would be *infinite*. Thus, high nuclear localization levels of NF-κB are overrepresented when measured with *NC* or *NCI*. On the other hand, if we assume that the total amount of NF-κB is constant within the time-span of our experiments (as in all mathematical models of the NF-κB signaling pathway [1], [2], [9], [10]) then the nuclear amount of NF-κB is directly proportional to *NT*; the total nuclear amount of NF-κB directs the transcriptional activity. For these reasons we use *NT* instead of *NC* and *NCI*, and we believe *NT* should be the standard quantifier of NF-κB dynamics.

### Section II: Results

#### II.A Performance of the Method and Non-Zero Basal Levels of Nf-κb

**Figure 4:**
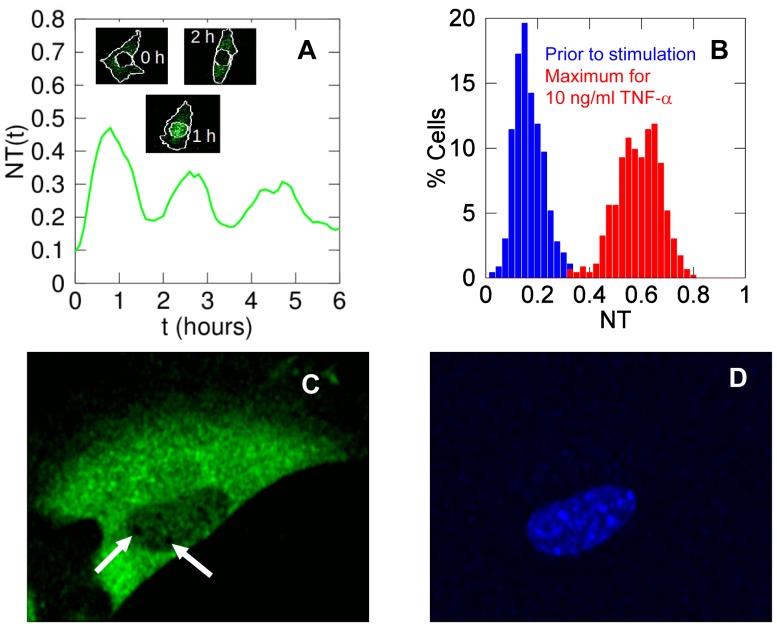
MEFs have non-zero nuclear basal levels of NF-κB. (**A**) A time series of a cell exposed to 10 ng/ml TNF-α; the insets show the nuclear and cytoplasmic segmentations at different times. (**B**) Distribution of the initial *NT* values in unstimulated cells (blue) and maximal NT values in cells stimulated with 10 ng/ml TNF-α (red): the blue histogram indicates that most unstimulated cells have low but non-zero levels of nuclear NF-κB. (**C**) Image at high magnification (63×) in the GFP channel of an optical slice from an unstimulated cell. The part corresponding to the nucleus shows fluorescence values clearly higher than the background. Nucleoli are the small black areas indicated by arrows. This is representative of cells for which the basal levels of NF-κB are well above the background. (**D**) Image of the same z-slice as in **C** acquired in the HOE channel confirms that we are observing an optical slice of the nucleus.

We have applied our method to 15 time-lapse microscopy experiments with GFP-p65 knock-in MEFs that were tracked for 15 hours. It is known that GFP tagging does not alter the biochemical activities of p65 and the expression of GFP-p65 is identical to that of p65 in wild-type MEFs, so our cells present physiological levels of p65/NF-κB [13]. Cells were stimulated with five different constant concentrations of TNF-α: 0, 0.1, 1, 10 and 100 ng/ml (three replicates per concentration, Materials and Methods). With our settings, about 30% of the cells are amenable to tracking for more than 3.5 hours. We excluded cells touching each other, moving out of the field, and the few undergoing mitosis or apoptosis. With the density of cells used, we tracked between 100 and 200 cells for 11 hours on average in each experiment. Importantly, the sizes of the cells are essentially identical in all the experiments indicating that the software performs consistently (see Table S1). Most cells stimulated with TNF-α do show repeated cycles of GFP-p65 re-localization between nucleus and cytoplasm. As an example, Figure 4A shows an NT time series for a cell stimulated with 10 ng/ml TNF-α; the insets show the segmentation of the nucleus and the cytoplasm at the indicated times. The segmentation works well both when GFP-p65 is in the cytoplasm and when it is mostly in the nucleus (see also Video S5).

The contribution of the cell autofluorescence to our estimation of GFP-p65 fluorescence is negligible. In fact, we did not collect any fluorescence signal from 3T3 cells plated with our GFP-p65 MEFs (see **Materials and Methods** and **Figure S1**).

When we estimated *NT* in GFP-p65 MEFs before stimulation (15 independent experiments, about 2000 cells) we found a non-zero basal value of *NT*  = 0.16±0.06 (mean ± SD). Approximately 85% of cells have *NT* ≥0.1 corresponding to 10% of NF-κB in the nucleus. The distribution of basal *NT* values is shown in **Figure 4B** (and **Table S1**).

In order to have a more direct and visual confirmation of the non-zero basal level of nuclear GFP-p65/NF-κB in unstimulated cells, we analyzed z-stacks of unstimulated cells at higher magnification. **Figures 4C and 4D** show one optical slice of a representative cell in the GFP and the HOE channel, respectively. After background subtraction, there is some GFP fluorescence co-localizing with the Hoechst staining, indicating that it does come from the nucleus. Moreover, it clearly delineates nucleoli (white arrows). The fluorescent signal coming from the cytoplasmic fraction above or below the nucleus is almost zero, excluding a possible contribution to the non-zero basal level (**Figure S2**). An example of a z-stack, in **Figure S3**.

It is possible to have an alternative estimation of *NT* by analyzing the “sum slice projection” of the z-stack [31] (see **Figure S2D**). By manually segmenting nuclei and cytoplasms in the blue and green channel, respectively, we get values of *NT*  = 0.15±0.06 (mean ± SD) which is very similar to the value we obtain with automated analysis.

We also calculated the *NT* value by segmenting nuclei and cytoplasms in z-stacks slice by slice (**Figure S3**). We did it for ten cells and got an *NT* value of about 0.11±0.04 (mean ± SD). This is somewhat smaller than the values we obtained using the automated analysis, indicating that the latter might slightly overestimate basal *NT*. Despite this possible systematic bias, we prefer the automated analysis, because in z-stack analysis the precision of cell segmentation is affected by inherent errors due to difficult identification of boundaries in planes with low fluorescence (top and the bottom of the cells; **Figures S2** and **S3**). Moreover, z-stack analysis exposes the cells to possible phototoxic effects and cannot be applied for long time-lapses. Reassuringly, both the z-stack and the automated approaches give values of *NT* significantly higher than zero, confirming the presence of non-zero basal levels of NF-κB in the nucleus.

In summary, we think that our method offers an adequate trade-off between low phototoxicity in long time lapses, high number of cells considered, high time resolution on one hand and good estimation of the value of *NT* on the other hand. This approach is suitable for cells where the contribution of the cytoplasm over and below the nucleus is minimal (e.g. fibroblasts and round cells like lymphocytes), but it might require minor modifications for those with a more cubic shape like epithelial cells.

Our results suggest that a minimal and sometimes transiently high localization of p65 is detectable in the nucleus of unstimulated cells. Data suggesting non-zero nuclear p65 levels have already been reported, obtained by imaging [1], [11], [20] or by ChIP among other assays (see i.e. Nowak et al, 2005 [21] and references therein). However, this feature has never been systematically evaluated or discussed. A basal p65 activity might be required for the maintenance of the repressed state of the NF-κB signaling through basal synthesis of IκB inhibitors, whose transcription is NF-κB dependent (see the **Discussion**).

#### II.B Descriptors of the Dynamics and Response to Stimuli

In our approach, the time evolution of NT is a cell type-independent quantifier of NF-κB dynamics. For this reason, NT can be used to unambiguously define NF-κB dynamical features in experiments with different cell types and stimuli, acquired on different days, and possibly from independent laboratories.

We have further characterized the *NT* quantifier using 5 descriptors: peak calling, fraction of responding cells, response time, maximum value and area under the response peak.

#### Peak calling

The negative feedback loop induced by the intrinsic time lag between p65 nuclear translocation, transcriptional activation and the accumulation of repressor proteins causes the NF-κB system to display oscillatory dynamics. Variations in nuclear p65 concentrations generate peaks that are the key descriptor we use for the dynamics of NF-κB in the following discussion.

**Figure 5:**
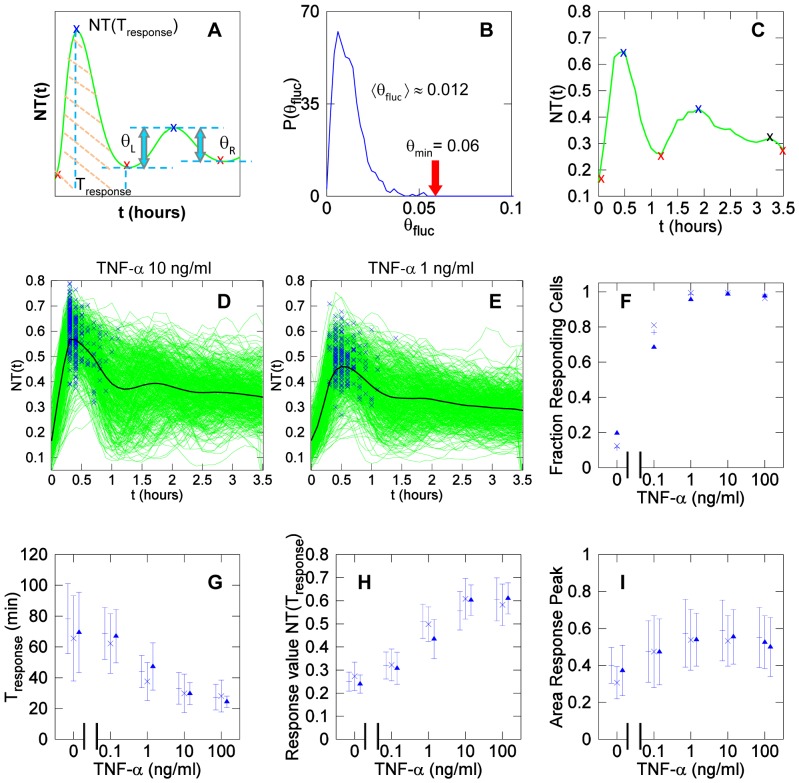
MEFs responses to TNF-α stimulation. (**A**) Graphical representation of a *NT* time series. A sequence of relative minimum (red ‘x’), maximum (blue ‘x’) and minimum identify a peak. The two quantities θ_L_ and θ_R_ are used to determine whether the second peak is significant. Other descriptors of the cell response to the stimulus are reported: timing of the response (T_response_), the response value NT(T_response_,) and the area under the response peak, defined as the area of the peak confined by its two local minima (shaded in orange). (**B**)The graph plots the probability of having a short-term fluctuation as a function of their θ value, θ_fluc_. Overall, short-term fluctuations have an average value of θ_fluc_ close to 0.012. From over more than 10^4^ short term fluctuations we find none with θ≥0.06. (**C**) Significant peaks (maximum and minima denoted by red and blue ‘x’ respectively) have θ≥0.06, other fluctuations are considered noise (black ‘x’). (**D**) Time series obtained with 10 ng/ml TNF-α and (**E**) 1 ng/ml TNF-α. Blue symbols represent the maximum of the first significant peak observed for each cell in the first 2 hours and denote a responding cell. The black line denotes the average of the time series, which looks strongly damped due to the asynchrony and heterogeneity of the dynamics at single cell level, plotted in green. (**F**) Fraction of responding cells as a function of TNF-α concentration. (**G**) Timing of the maximum of the response peak after stimulation as a function of TNF-α concentration. (**H**) The maximal value of the response peak increases with TNF-α doses and appears to plateau at 10 ng/ml. (**I**) The area under the response peak is remarkably constant with increasing TNF-α doses. In panels from (**F**) to (**I**), each symbol corresponds to an independent experiment; error bars represent standard deviation.

In an *NT* plot as a function of time (*NT(t)*), we define as a peak a sequence of a local minimum, a local maximum and a local minimum (red and blue “x” in **Figure 5A**). The parameters θ_L_ and θ_R_ describe the *NT* variations between a consecutive minimum and maximum and between the same maximum and the next minimum, respectively, as shown in **Figure 5A**. The peak value θis defined as the average between θ_L_ and θ_R_, or θ =  (θ_L_+ θ_R_)/2.

To be reliable descriptors, peaks have to be distinguished from intrinsic random fluctuations. On the other hand, a too restrictive selection of the peaks might imply the loss of some relevant information. For this reason it is important to define a threshold for the experimental noise. Experimental noise is expected to be responsible for the fast (high frequency) fluctuations of *NT*, defined as a sequence of a local minimum, a local maximum and a local minimum occurring in three consecutive frames (12 minutes). The distribution of these fluctuations in **Figure 5B** shows that their average value is close to θ = 0.01 in our experimental set-up, and the probability of finding fluctuations with θ≥0.06 is less than 10^−4^, which means none found in approximately 10^4^ such short term fluctuations in our data. For this reason we select θ_min_  = 0.06 as a robust threshold above which a peak is considered significant. From now on, a *significant peak* has θ_L_≥θ_min_ and θ_R_≥θ_min_ and obviously a peak value θ≥θ_min_. We wrote a simple algorithm (**Document S3**) to detect in time series significant peaks representing NF-κB dynamics and discard fluctuations due to noise (**Figure 5C**).

#### Responding cells and response peak

The definition of peaks allows the description of a cell that responds to an inflammatory stimulus. Therefore we define as “responding cell” a cell showing the first significant *NT* peak (θ≥0.06) in the first 2 hours after TNF-α stimulation. Such peak will be referred to as the *response peak* (see below).

*NT* time series were analyzed to quantify the fraction of responding cells for different concentrations of TNF-α. We used time series truncated at 3.5 hours and nearly all the cells had one significant peak (*response peak*) in the first 2 hours after stimulation for TNF-α ≥1 ng/ml. Examples of oscillatory dynamics for TNF-α concentrations of 10 ng/ml and 1 ng/ml are given in **Figure 5D and 5E**, respectively, together with their average curves (green and black lines, respectively). Significant peaks are marked with blue symbols. The fraction of responding cells increases with increasing TNF-α concentrations and plateaus for concentrations above 1 ng/ml as shown in **Figure 5F** **,** in accordance with previous observations [10].

#### Response time

The response time *T_response_* is given by the position of the response peak on the time axis (see **Figure 5A**). For high TNF-α doses such as 10 and 100 ng/ml the response is fast (approx. 30 minutes) and very synchronous in the cell population (low Standard Deviation in **Table 1** and **Figure 5G**). For lower concentrations, the cells are slower to respond and more asynchronous.

**Table 1:** Parameters of the response.

(ng/ml)	Fraction responding	*T_response_(mins)*	NT(*T_response_*)	Area Peak
**0**	0.15 (0.05)	70 (30)	0.25 (0.05)	0.36 (0.11)
**0.1**	0.75 (0.06)	64 (17)	0.32 (0.07)	0.48 (0.19)
**1**	0.98 (0.02)	42 (13)	0.48 (0.08)	0.55 (0.14)
**10**	0.991 (0.004)	31 (11)	0.59 (0.09)	0.56 (0.14)
**100**	0.98 (0.01)	26 (8)	0.60 (0.08)	0.52 (0.16)

Mean values of the parameters describing the response of the cells to the stimulus for each dose (Standard Deviation is calculated from 3 independent experiments and given in parenthesis). Column headers: **Fraction responding**: fraction of cells with a significant peak in the first two hours after the stimulus: ***T_response_***:timing of the response. **NT(** ***T_response_*** **)**: value of the response peak. **Area Peak**: area of the response peak.

#### Response maximum

The response maximum is the maximal *NT* value of the response peak *NT(T_response_)(* **Figure 5A**).The response maximum increases with TNF-α concentration and plateaus close to 0.6 for 10 ng/ml TNF-α (**Figure 5H**), which is higher than the value reported by others for higher stimulation doses [22]. This implies that on average 40% of NF-κB remains in the cytoplasm even upon maximal stimulation (**Table 1**).

Since we have assessed the relationship between *NT* and the Nucleus-to-Cytoplasmic ratios (*NC* and *NCI* see **Document S2**) as used in previous works [1], [9], [11], [12], [19], we can compare our results to what has been published. As we can see in **Figure 3**, an *NT* value variation in the range 0.5 to 0.75 leads to *NC* values in the range 1 to 3 and even 3-6 depending on the computing procedure (see also **Document S2**). Overall, then, our results are broadly compatible with those reported by others [1], [9], [11], [12], [19]. For the reasons pointed out in **Section I.B**, we believe that the *NT* quantifier is a univocal tool for a more direct comparison of data generated in independent laboratories.

#### Area under the response peak

Finally, we calculate the area under the response peak as the area between the two local minima that frame the response peak as we show in **Figure 4A** (peak area with light red lines). The area under the peak estimates how much NF-κB goes inside the nucleus and for how long, the two parameters that presumably are dominant in gene regulation. **Figure5 I** shows that the area under the response peak has quite similar values for various TNF-α concentrations and standard deviations are also similar. This phenomenon has been referred to as *digital activation* and indicates that the early transcriptional response is quite independent of the stimulus dose [10].

A summary of the features of the response descriptor for each TNF-α dose is given in **Table 1**. Overall, our approach identifies a panel of descriptors for an unbiased and meaningful comparison of NF-κB dynamics obtained from different cell types, upon different stimuli and in different experimental conditions.

#### II.C Spontaneous Nf-κb Activity

**Figure 6:**
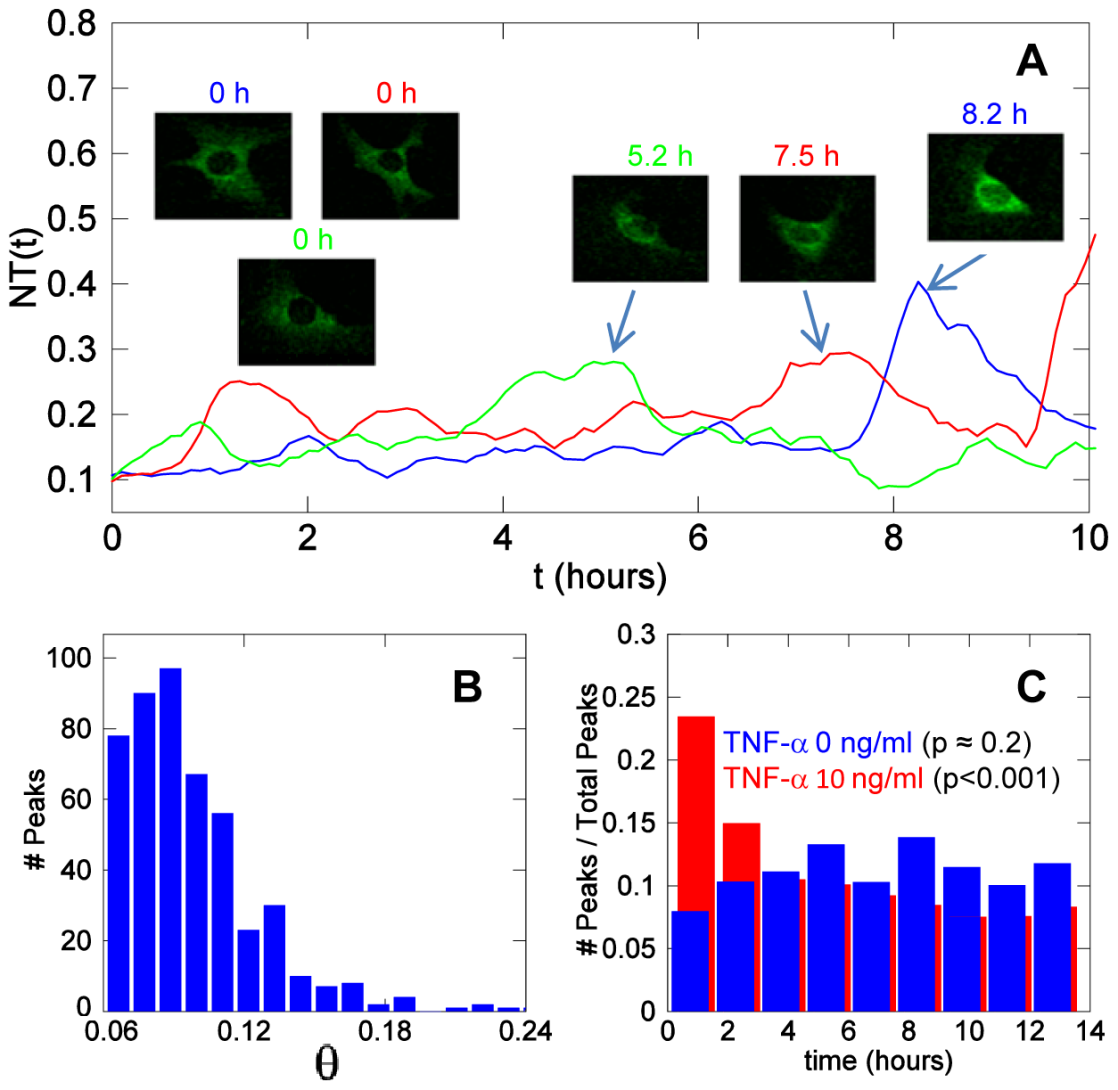
Spontaneous NF-κB dynamics in the absence of stimuli. (**A**) Three *NT* time series corresponding to three unstimulated cells showing spontaneous activity. The insets show the cells corresponding to the traces (same color code) at time zero and at the maximum of the most conspicuous peaks. (**B**) Distribution of the peak values observed in unstimulated cells (out of 300 cells tracked, see **Table S1**). (**C**) The distribution of the timing of the peaks observed for unstimulated cells (blue) and for cells stimulated with 10 ng/ml TNF-α (red).The *p*-values are for the null hypothesis “the peaks are evenly distributed (chi-square test with 8 degrees of freedom).

According with our descriptors, a small fraction of unstimulated cells can be classified as responding (see Figure 5 F and Table 1). In fact, 15% of the cells show a significant peak with NT = 0.25±0.05 during the first two hours of the experiment, which hints to the existence of spontaneous activation of NF-κB signaling. Over 15 hours, as many as 70% of the cells show spontaneous significant peaks. Three NT time series for three unstimulated cells displaying large spontaneous peaks with θ ranging from 0.1 to 0.24 are shown in Figure 6A (see also Video S6). The distribution of θ values for spontaneous peaks is reported in Figure 6B. The mean is close to 0.1. The average frequency of spontaneous peaks per cell is 0.11/hour (i.e., one peak every 9 hours).

In **Figure 6** **C** we show the distribution along time of the peaks detected in both unstimulated (blue bars) and TNF-α stimulated cells (red bars, 10 ng/ml). The peaks in unstimulated cells are evenly distributed over time while those in stimulated cells are clustered between 0 and 2 hours post-stimulation. The null hypothesis that “the peaks are evenly distributed in time” cannot be rejected for unstimulated cells (*p*≈0.2, chi-square test), whereas it can be rejected for stimulated cells (*p<*0.001). Small *p*-values are also observed when a similar test is run for cells stimulated for other TNF-α concentrations (see **Figure S4**).

Although it has been shown that NF-κB can be activated even at very low doses of TNF-α [19], as suggested by mathematical models of the system [23], to our knowledge this is the first direct evidence with statistical significance of the presence of spontaneous activation of NF-κB. Spontaneous activity has also been observed for other transcription factors such as p53 [24]. Determining whether these spontaneous activations are related with internal processes of the cell or triggered by external events would require further exploration.

#### II.D Dynamics of Nf-κb upon Tnf-Α Stimulation

**Figure 7:**
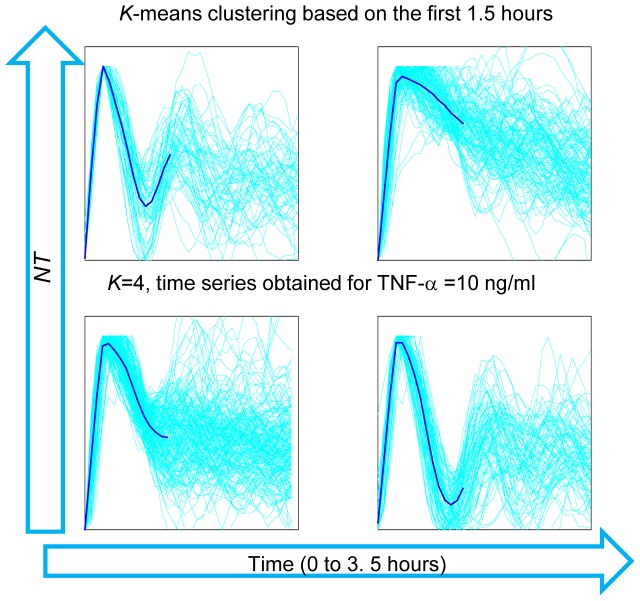
Clustering analysis of the short-term dynamics of NF-κB. Clustering of the *NT* traces for the first 1.5 hours of cells exposed to 10 ng/ml TNF-α, using a *K*-means algorithm. The individual traces (450) are in cyan and the centroids of the cluster are plotted in blue.

The oscillatory dynamics of cells upon stimulation is quite heterogeneous in our hands, as compared to observations from other groups where oscillations where typically observed as a high peak followed by smaller peaks. In order to have an unbiased assessment of this heterogeneity, we clustered the NT(t) profiles obtained with 10 ng/ml TNF-α using κ-means clustering. Figure 7 shows four clusters of the time series truncated at the first 3.5 hours and based on the profiles in the first 1.5 hours. This clustering reveals a high heterogeneity in the dynamics even for this short time. In particular, we can see that for a significantly high number of trajectories the values of the second peak of NT are close and even higher than the values of the response peak.

**Figure 8:**
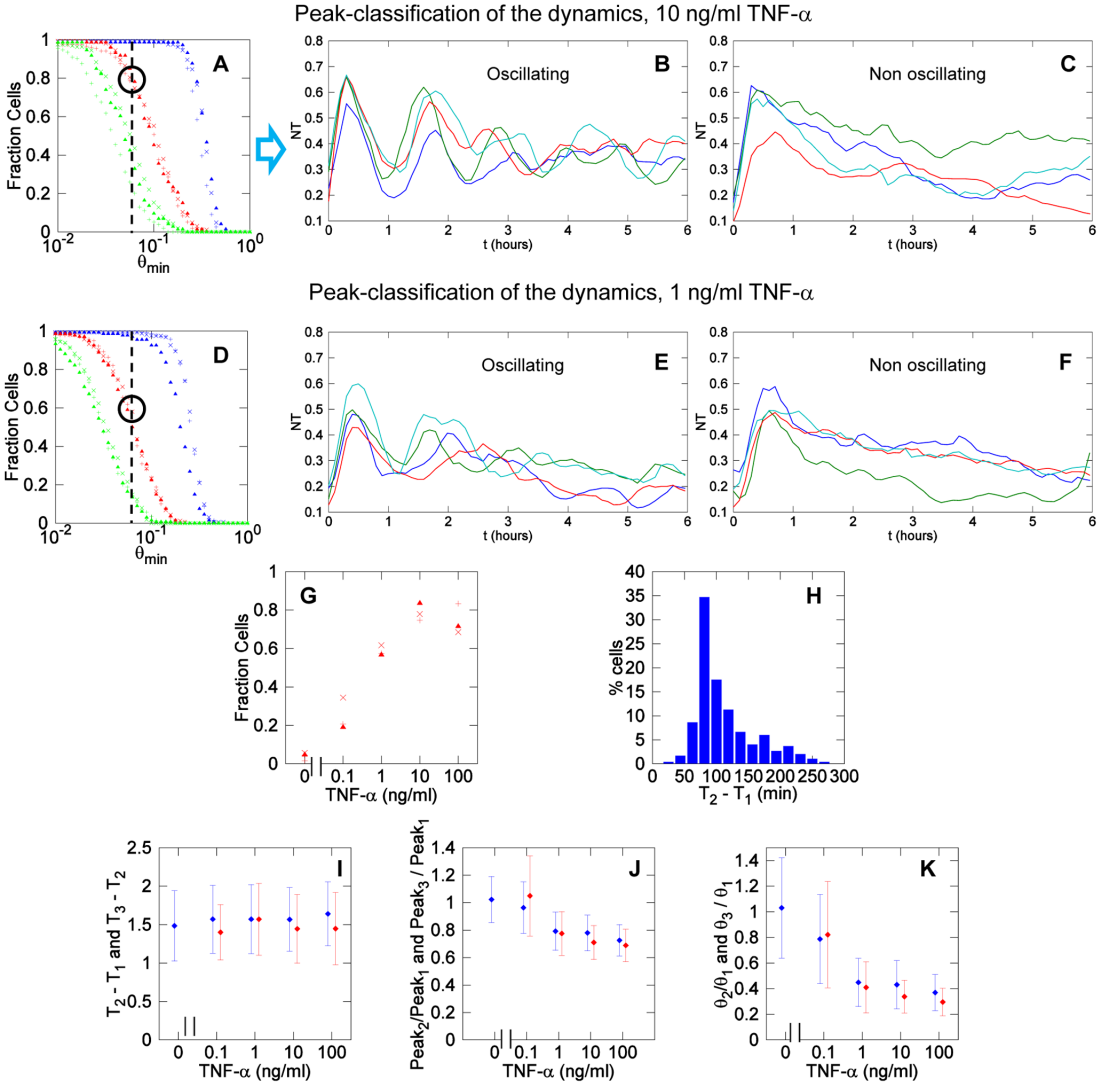
Heterogeneous dynamics of NF-κB. (**A**) Fraction of cells exposed to 10 ng/ml TNF-α that show at least one (blue), two (red) and three (green) peaks as a function of the threshold θ. Each kind of marker is representative of a single experiment. The light black dotted line indicates the threshold above which a peak is significant using our criterion. For θ≥0.06 the fraction of cells having at least two peaks (oscillating cells) is close to 0.8. (**B**) Examples of oscillating and (**C**) non-oscillating cells for 10 ng/ml TNF-α. (**D**) Fraction of cells exposed to 1 ng/ml TNF-α having one (blue), two (red) and three (green) peaks as a function of the threshold θ. (**E**) Examples of oscillating and (**F**) non-oscillating cells for 1 ng/ml of TNF-α. (**G**) Fraction of oscillating cells (with at least two peaks with θ>0.06 in the first 5 hours) for different concentrations. Each marker represents results from an independent experiment. (**H**) Histogram of the time interval between the first two peaks for TNF-α 10 ng/ml; the mode is close to 90 minutes. (**I**) The time interval between the second and the first peak (blue) and the third and the second peak (red) is remarkably constant and independent from the dose of TNF-α. (**J**) Ratio of the values of the second and first peak (blue) and of the third and first peak (red) for oscillating cells. The values are close to 1, indicating that oscillatory peaks are similar in height. (**K**) Ratio of the second and first peak values θ_2_ and θ_1_ (blue) and of the third and first peak values θ_3_ and θ_1_ (red) for oscillating cells. Second and third peaks tend to have smaller peak values.

Our definition of peaks provides as a corollary a simple definition of oscillatory behavior as the occurrence of more than one significant peak in a single cell during a certain period. In **Figure 8A** we plotted the fraction of cells having one (blue), two (red) and three peaks (green) within 5 hours after stimulation as a function of θ. The coincidence between the results of the three biological replicates is quite remarkable. Considering the significant threshold of θ>0.06, approximately 80% of the cells do oscillate, in accordance with our previous work [9]. In **Figure 8B** we show examples of typical time series of oscillating cells while **Figure 8C** shows examples of non-oscillating cells (see also **Video S7 and S8**).

We used the same tools for all the concentrations of the stimulus considered. **Figure 8D** shows the fraction of cells stimulated with 1 ng/ml of TNF-α having one, two and three peaks as a function of θ. Again, the results obtained for three experiments are very similar. With our definition of oscillating cell, we find that roughly 60% of the cells do oscillate when stimulated with 1 ng/ml TNF-α. Examples of oscillating and non-oscillating cells are shown in **Figure 8E and F**, respectively (top and bottom, see also **Video S9** and **S10**).

These computations allow us to systematically show that the fraction of oscillating cells increases for increasing stimulus concentrations, and apparently saturates close to 0.8, as shown in **Figure 8G** **and** **Table 2**. Therefore, under our criteria, roughly 80% of GFP-p65 MEFs can oscillate. Still, even for high TNF-α concentrations about 20% of cells do respond with a first peak but do not oscillate, hereafter “non-oscillating” cells. At the opposite extreme of the spectrum, a fraction of oscillating cells exists in the unstimulated population, confirming the existence of spontaneous NF-κB activity.

**Table 2:** Parameters of the oscillations.

(ng/ml)	Fraction oscillating	*T_2_ – T_1_ (mins)*	Peak 2/Peak 1	*T_3_ – T_2_ (mins)*	Peak 3/Peak 1
**0**	0.04 (0.02)	89 (27)	1.0 (0.2)	X	X
**0.1**	0.25 (0.05)	94 (27)	0.96 (0.19)	84 (22)	1.0 (0.3)
**1**	0.59 (0.03)	94 (26)	0.79 (0.14)	94 (28)	0.8 (0.16)
**10**	0.79 (0.04)	94 (25)	0.78 (0.13)	87 (27)	0.71 (0.12)
**100**	0.74 (0.08)	98 (25)	0.73 (0.11)	87 (28)	0.69 (0.12)

Mean values of the parameters describing the oscillating behavior for each dose (standard deviation calculated from 3 independent experiments and given in parenthesis). Column headers: **Fraction oscillating**: Fraction of oscillating cells. ***T_2_ - T_1_***: timing between the second and the first peak. **Peak2/Peak1**: ratio between the NT values of the second and the first peak. ***T_3_ – T_2_***: timing between the third and the second peak. **Peak3/Peak1**: ratio between the NT values of the third and the second peak.

An additional relevant feature of oscillation is the period, that can be estimated as the interval between successive times of the peaks, noted {T_n_} (n = 1, 2, …). **Figure 8H** plots the distribution of the intervals between peaks in the cell population for 10 ng/ml of TNF-α. We have computed T_2_ –T_1_ and T_3_ –T_2_ for different concentrations (**Figure 8I**) and found that the oscillations period is robust and independent of TNF-α concentration, as previously reported [1], [11]. To confirm our results we have performed a spectral analysis of the time series. The average periodograms obtained for all the doses of TNF-α (see **Figure S5**) confirm an average period of 1.5 hours; this is more evident when the spectrum is computed considering only oscillating cells. We find no evidence of longer or shorter periodicities in the oscillations.

Finally, another feature worth exploring is the dampening of oscillations. **Figure 8J** shows the ratio between the second or the third peak maxima and the first response peak. Ratios between the second and the response peak range from 0.7 to 1 for all TNF-α doses, and the same values are found for the third peak, values sensibly higher than those reported by others [1], [10], [11]. Sometimes the second and the third peaks are even higher than the first peak (this is particularly clear in the clustering shown in **Figure 7** and in some time series displayed in **Figure 8B and E**). On the other hand, if we look at the ratios of the peak values of the second and the third peak (θ_2_ and θ_3_) with the peak value of the first one (θ_1_ **,** **Figure 8K**), we find that these ratios decrease with the dose. Taken together, these two observations suggest the presence of values of *NT* consistently higher than the basal level after the response peak. This is illustrated in **Figure S6**. Overall we conclude that for many cells oscillations are not as strongly damped as previously reported, although the dynamics observed is strongly heterogeneous which might make the population response more robust [25]. Finally, we have found that the area values from the second and third peaks are on average also remarkably similar in spite of the shape heterogeneity (see **Figure S7**). Thus, digital activation features are also observed in the second and third cycle of translocation.

**Figure 9:**
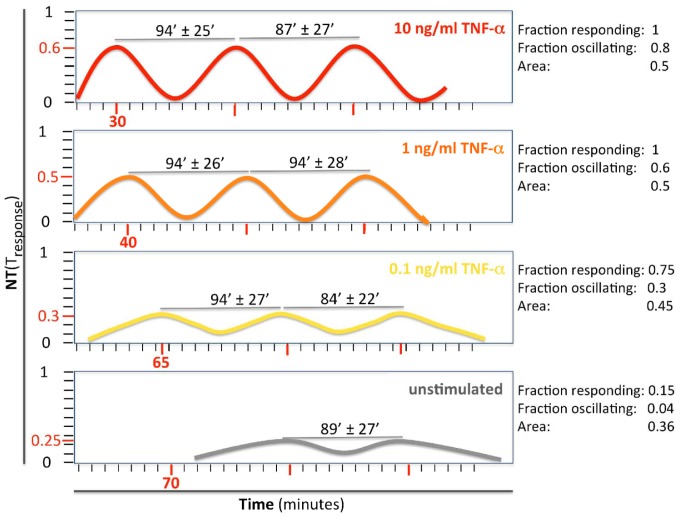
Schematic representation of the oscillatory features of NF-κB upon decreasing TNF-α stimulation (from 10 ng/ml, red curve, to 0 ng/ml, gray curve). On the x-axis (time), red numbers and ticks mark the mean period of oscillations that is specific for each stimulation, in minutes. On the y-axis, the mean *NT* value specific for each stimulation is indicated in red. Numbers over the gray lines indicate the mean interval in minutes between two consecutive peaks (T_2_-T_1_ and T_3_-T_2_ as reported in Table S1). On the right side, mean values of responding and oscillating cells are reported for each condition. The mean area values are also indicated. It is possible to appreciate that the period is constant and peaks have a minimal variation in height.

We provide a summary of the dynamical features observed for each TNF-α dose in **Table 2** and **Figure 9**.

## Conclusions and Discussion

NF-κB is the pivotal transcription factor that controls multiple steps of inflammation, from microbial killing to endothelial activation and cell migration, through the co-ordinate transcriptional activation of hundreds of genes. The degradation of the IκB repressors followed by the nuclear localization of NF-κB and the delayed re-synthesis of the IκB repressors accounts for the negative feedback loop in the system and prompts it to display oscillatory dynamics. Oscillatory dynamics have been described by several labs but it is not clear so far whether it is an unavoidable consequence of the design of the system or it has adaptive advantage. To start addressing this question a quantitatively accurate description of NF-κB dynamics is essential.

We developed a method to automatically analyze NF-κB dynamics in hundreds of cells, and to correct for image distortions that traditionally had been overlooked. We then defined adequate quantifiers to describe the oscillatory dynamics; these can be applied to analyze and compare the results from different experimental setups.

In our setup, we took advantage of the well characterized GFP-p65 knock-in cell system expressing physiological amounts of p65 [9], [13]. Of note, in these cells the molecular stoichiometry of the NF-κB signaling molecules is safeguarded, contrary to what may happen in transiently or stably transfected cells.

### Image acquisition

Fluorescence intensities can be matched to protein amounts only if the image thickness is sufficient to capture the signal emitted from the whole cell and if the image background is subtracted. To abide by this rule, we captured the total amount of p65 per cell using a single-exposure image taken with an open pinhole. This technical detail also avoids extensive bleaching and photo-damage that leads to NF-κB activation, possible events when the alternative method of z-stack acquisition is applied.

### Background estimation

We found that the background varies in different regions of the same image and along the timeline, due to optical distortions, variations in the efficiency of the light detectors or uneven illumination, e.g. fluctuations of the laser source.

A careful quantification of background in the immediate surroundings of the cell improves the signal-to-noise ratio. Moreover, the assignment of pixels to the background or to the cell allows a precise segmentation of the cytoplasm boundaries.

Although the whole procedure is computationally heavy, the reward is high and we obtain measures that are free of the distortions inherent to most of time-lapse microscopy experiments.

### I. Quantifier and Descriptors

*NT value.* We chose the *NT* ratio as the most significant quantifier for NF-κB dynamics, as opposed to the commonly used *NC* ratios. *NT* ratio is a cell type-independent and internally normalized parameter that accurately represents p65 re-distribution upon stimulation. It also avoids approximations due to changes in cell shape. Of note, it can vary between 0 and 1, therefore datasets from different labs or set-ups can be easily compared. The use of *NT* allows us to define accurate descriptors of the dynamics observed for different stimuli.

#### First response upon TNF stimulation

We verified that the intensity of the stimulus tunes the timing and the intensity of the first response: the higher the TNF-α concentration, the faster, more synchronous and more intense the response is in the cell population (Table 1 and 2), in accordance with previous observations [10] .

*NT* ratios revealed a previously unappreciated feature: p65 translocation into the nucleus is never complete. In fact for TNF-α ≥1 ng/ml, approximately 40% of the molecules are detected in the cytoplasm during the first round of translocation. This can simply indicate that only a fraction of molecules are committed to translocate, or that there is a basal nucleus-to-cytoplasm transport in the time scale of minutes [9]. The area under the first peak is found to be independent of the dose of TNF-α, a phenomenon that has been referred to as “digital activation” because it correlates with the observed dose-independent gene transcription of early genes at single cell level [10].

#### Dynamics upon TNF-α Stimulation

We assigned clear definitions to traditionally imprecise notions, such as responding cell and oscillating cell. We confirmed that around 80% of GFP-p65 MEFs do oscillate at 10 ng/ml TNF-α [9]. The reason why a constant fraction of cells do respond to TNF-α but do not oscillate still needs to be investigated.

We found striking similarities and unexpected differences in the overall profiles deriving from different TNF-α stimulations, summarized in the cartoon in **Figure 9**. The fraction of responding cells varies approximately from 100 to 75% over 3 orders of magnitude of stimulus intensity. Instead, the fraction of oscillating cells varies between 80 and 25%. Therefore, most cells respond while most of the variability is in the number of oscillating cells.

The ratios between the *NT* maxima of any of the peaks and the *response peak* are quite close to one, and thus the system is not strongly dampened, contrary to what was previously indicated [2], [10], [11]. Dampening might have been overestimated because the Nucleus-to-Cytoplasmic (*NC*) ratio has been frequently used. In fact, the relationship between *NT* and *NC* is nonlinear as reported in **Figure 3** (i.e. *NT* = 0.8 corresponds to *NC* = 4 while *NT* = 0.4 corresponds to *NC*≤1.3), and a small difference in nuclear content is translated into a wide variation in *NC*. This affects the first peak more than the following ones. The presence of dampening might also be related to the biological system used. The period of the oscillations has an average value of approximately 90 minutes. Of note, this parameter is remarkably constant even for different stimulation intensities. This observation might suggest that the NF-κB oscillatory system is programmed to respond with a specific frequency [10]. In spite of the observed heterogeneity in the second and third peak of the oscillations, we find that the area under these peaks is also remarkably constant for all the doses of TNF-α used, indicating that some features of the digital response might be conserved in subsequent oscillatory cycles.

#### Heterogeneity of the response and the dynamics

We have found relatively small variability for the response parameters. However, the clustering of NT traces shows heterogeneity in the shape of the first peak for 10 ng/ml TNF-α (Figure 7). In particular, the features of the response peak in the first 1.5 hrs upon stimulation do not determine the dynamics for subsequent times. There are different possible sources of this heterogeneity. A recent study suggests that the delay between the synthesis of the inhibitor IκBα and IκBε maximizes the heterogeneity, which might be an advantageous feature leading to population robustness [25]. Others have shown that different IKK activation profiles can give rise to different dynamics, as suggested by mathematical models [26]. Furthermore, mathematical models suggest that the activation of TNFR1 receptors via TNF-α and activation of A20 and IκB genes are the key sources of stochasticity in the system [23]. The mounting evidence on the stochasticity of gene expression [27] suggests that the latter can play a particularly relevant role. The negative feedback present in the pathway, embodied in the synthesis of IκB repressors, is transcription-based and is therefore essentially a stochastic process; some realizations of this process might imply the absence of oscillations. To clarify this point, the use of simple mathematical models of the NF-κB system might be of help [28].

### II. Novel Results: High-Throughput Quantification of Non-Zero Basal Level and Spontaneous Nf-κb Activation

Our high-throughput analysis has identified the presence a non-zero basal level of p65 in unstimulated cells. Moreover, we have identified spontaneous activation of NF-κB system.

Approximately all the cells at basal state have an NT value ≥0.1 while at least 15% of cells show also a response peak in the first 2 hrs.

When longer time lapses are considered, spontaneous peaks are uniformly distributed in time, whereas some peaks occur in doublets spaced by approximately 90 min, which is the spacing observed following stimulation.

Basal nuclear localization and spontaneous activation can be the two sides of the same coin. We believe that the basal level of NF-κB localization is necessary to keep the system from firing, as some nuclear NF-κB is necessary for IκB repressor transcription. Indeed, the IκB-α gene contains as many as 6 NF-κB binding sites [29] which makes it much more sensitive to the level of nuclear NF-κB than other genes that generally contain 2 or 3 NF-κB binding sites. IκBα, as any other protein, is subjected to turnover, and needs to be replenished continually to prevent the spontaneous activation of the NF-κB system. If the Iκβ-α protein level decreases too much, NF-κB will translocate to the nucleus in larger amounts and start a spontaneous cycle of activation. Some flipping of the IκBα conncentration below the minimum required to keep the system from firings is predicted, considering the stochastic and discrete nature of gene expression [27]. An alternative hypothesis is that the spontaneous firings are due to events occurring in the vicinity of the cell, which matches well with the experimentally observed ability of NF-κB to respond to low doses of the stimulus [19], as suggested by mathematical models [23]. Investigating which of these two hypotheses is the one leading to spontaneous activation of NF-κB making use of our methodology is a natural continuation of the present study.

In conclusion, we have re-evaluated NF-κB oscillations using a new signal detection method and a more clearly defined conceptual framework. Our high-throughput analysis integrates many features previously observed by different labs, but also indicates that there is a basal activity of NF-κB in most cells, and a spontaneous random firing of the NF-κB system in a small fraction of cells at any time, but in a large fraction of cells over long times. This predicts that a localized, low-level synthesis of inflammatory molecules may occur in non-stimulated tissues (consisting of billion of cells) possibly providing an autocrine/paracrine activation. Such a prediction will need a careful experimentation for confirmation or refutation.

## Materials and Methods

### Cell Culture and reagents

GFP-p65 knock-in fibroblasts, a kind gift of M. Pasparakis [13], were cultured in phenol-red free DMEM, supplemented with 10% FCS, 50 µM β-Mercaptoethanol, 1% L-Glutamine, 1% Sodium Pyruvate, 1% non-essential amino acids, and Pen/Strep. Cells were plated on coverslip-bottom dishes (MatTek, Ashland, MA) 24 hrs before imaging and the medium was changed to DMEM-0.1% FCS 6 hours before imaging. TNF-α (R&D Systems) was diluted in DMEM-0.1%FCS and injected in the well to a final concentration of 0.1, 1, 10 and 100 ng/ml. In order to estimate autofluorescence, GFP-p65 knock-in MEFs were plated together with NIH-3T3 cells following the culture procedure described. For nuclear staining, Hoechst 33342 was added 1 hour before the experiment at 50 ng/ml concentration.

### Time-lapse Microscopy experiments

Live cell imaging of GFP-p65 knock-in MEF was performed using a Leica TCS SP5 confocal microscope with an incubation system where cells were stably maintained at 37°C with humidified 5% CO_2_. Time-lapse images were acquired at 6 min intervals during 15 hours. Using a low magnification objective (20×, 0.5 NA) and an open pinhole (Airy 3), the image width (10.7 µm) contains the thickness of the whole cell. GFP-p65 is imaged at 488 nm while Hoechst 33342 stained nuclei are imaged at 405 nm. TNF-α (R&D Systems, Minneapolis, MN) was applied at the second time point by gentle injection through a tubing. Images were acquired as 16 bit, 1024×1024, TIFF files. Experiment replicates were acquired on different days starting from different batches of frozen cells.

Transmission images are acquired with 488 nm laser to identify non-fluorescent NIH-3T3 cells co-plated with GFP-p65 MEFs.

To have a visual confirmation of the non-zero basal levels of NF-κB of the nucleus, we acquired z-stacks of unstimulated cells with a 63× Plan-Apochromat oil immersion objective (1.4NA), pinhole = Airy1. Zoom 6×, Z-width:500 nm.

### Software

The software implementation of our method was run in GNU Octave, version 3.2.4, compatible with MATLAB. The program *tiffread.m* [30] was used for converting the TIFF images obtained from the time-lapse microscopy into real matrices.

A detailed description of the software is provided in **Documents S1-S3.**

Image files from the Leica system (.lif) have been handled with ImageJ that has also been used to produce “Sum slice” projections [31].

## Supporting Information

Figure S1 **Cell autofluorescence has a negligible effect.** NIH-3T3 and GFP-p65 MEFs are imaged in the GFP channel **(A)** to detect green fluorescence and in the transmission channel to detect all the cells in the field **(B)**. In **C** is shown a different representation of **A** using a pseudo-color scale where high GFP intensities are extremely compressed (white for values from 4.000 to 20.000) while the signals in the lower range that approximate the background are represented on an extended scale and allow to appreciate minimal increases (orange). 3T3 cells are not observable in the GFP channel so we conclude that contribution of autofluorescence to our estimation of *NT* is negligible.
(TIF)

Figure S2 **Additional data to support the existence of nonzero basal levels of nuclear NF-**κ**B.** Cell reconstruction using the z-stacks (see **Materials and Methods**) in **(A)** the HOE channel, **(B)** the GFP channel and **(C)** both, merged. We can see that the contribution of the cytoplasm above and below the nucleus is very small. Segmenting the “Sum slice” projection shown in **(D)** we obtain values of NT(0) = 0.15±0.06, compatible with our automated methods (mean and standard deviation computed for 30 cells).
(TIF)

Figure S3 **Example of a z-stack performed on unstimulated GFP-p65 MEFs.** Z-stacks have been acquired with a 63x obj. and a z-width of 500 nm. Each cell has been segmented in the Hoe and the GFP channels to quantify the GFP fluorescence in nuclei and in the whole cells in each plane of the z-stack. By summing nuclear and cytoplasmic intensities from the whole stack for all the cells we get a value NT(0) = 0.11±0.04 (mean and standard deviation computed for 10 cells). Note that this segmentation is affected by inherent errors due to the imprecise identification of boundaries in planes with low fluorescence (top and the bottom of the cells; see also **Figure S2**). Moreover, z-stack analysis exposes the cells to possible phototoxic effects and cannot be applied for long time-lapses. The provided z-stack file is in tiff format and can be opened in ImageJ. Green and blue channels can be independently regulated to appreciate the contribution of each component.
(TIF)

Figure S4 **The response peak occurs in the first 2 hours after stimulation.** Distribution in time of the significant peaks observed for cells using different stimulations (e.g. for 100 ng/ml TNF-α, 25% of peaks are in the first 2 hrs). When applying a test for uniformity of the timing, we always get *p*-values<0.001.
(TIF)

Figure S5 **Spectral analysis of the time series confirms a T = 1.5 h periodicity in the dynamics. (A)** Average periodograms for the time series obtained for the different doses used. In the periodograms we plot the amplitude of the Fourier transform for each period T. Average for **(B)** only oscillating cells and **(C)** non-oscillating cells for the doses of TNF-α considered. Thicker lines correspond to higher doses. Periods close to T = 1.5 hours are enhanced, and this enhancement is more evident for oscillating cells. The high period (low frequency) enhancement is due to the dampening observed for the average of the time series. **(D)** Sinusoidal time series **(E)** superposition of a high and low frequency sinusoidal time series and **(F)** series of an exponential decay. The time series have the same number of points that the series used for the periodograms. In **(G)**, **(H)** and **(I)** we show their respective periodograms. The pure frequencies can be easily discerned. On the other hand, the high period (low frequency) enhancement observed in the periodogram of the exponential decay can be related with the one observed in the average periodograms of the time series of *NT*: which correspond to the one that would be observed for the damped average time series.
(TIF)

Figure S6 **An example of a time series with high Peak_2_/Peak_1_ and lower.** θ**_2_/**θ**_1_.** The ratio between the second and the first peak heights (Peak_2_/Peak_1_) is close to 0.8. The peak values of each peak are calculated as θ_i_ = 0.5·(θ_L,i_+ θ_R,i_), for the first peak θ_1_ it is close to 0.3 and for the second θ_2_ is close to 0.15, so the ratio between the peak values is close to 0.5. This situation is common in our time series and thus in general θ_2_/θ_1_ is smaller than Peak_2_/Peak_1_.
(TIF)

Figure S7 **Area values from the second and third peaks.** Area values under the second and the third peaks (when observed) are plotted for different TNF-αconcentrations. On average, the area values are remarkably constant.
(TIF)

Table S1 **For each experiment performed we show the number of cells observed, the % selected, the average tracking time, the area (in pixels) of the nuclei and the** ***NT*** **value observed at time 0.** We see that for all the experiments there is a nonzero basal level of nuclear NF-κB. Results are given as mean (standard deviation).
(DOCX)

Document S1 **Description of the software used for the single-cell quantification of NF-**κ**B dynamics.**
(DOC)

Document S2 **Relation between different quantifiers of NF-**κ**B dynamics used in the literature.**
(DOC)

Document S3 **Pseudocode for the detection of significant peaks.**
(DOC)

Video S1 **Time-lapse movie of our cells.** The HOE channel for cells stained, as described in Materials and Methods. Total time for Videos S1 to S4 is 15 hours.
(AVI)

Video S2 **Time-lapse movie of our cells.** The GFP channel for cells stained, as described in Materials and Methods.
(AVI)

Video S3 **Time-lapse movie of our cells.** The HOE channel for non-stained cells, as described in Materials and Methods.
(AVI)

Video S4 **Time-lapse movie of our cells.** The GFP channel for cells non-stained, as described in Materials and Methods.
(AVI)

Video S5 **Nucleus and cytoplasm segmentation.** Example of the segmentation of nucleus and cytoplasm obtained from time-lapse data of GFP-p65 MEFS stimulated with 10 ng/ml TNF-α. Cells detected but not tracked are marked with an ‘x’, knowing their approximate position is useful for the background reconstruction.
(MP4)

Video S6 **Example of the spontaneous activation of NF-**κ**B observed in an unstimulated cell.**
(MP4)

Video S7 **Example of an oscillating cell upon 10 ng/ml TNF-α stimulation.**
(MP4)

Video S8 **Example of a non-oscillating cell upon 10 ng/ml TNF-α stimulation.**
(MP4)

Video S9 **Example of an oscillating cell upon 1 ng/ml TNF-α stimulation.**
(MP4)

Video S10 **Example of a non-oscillating cell upon 1 ng/ml TNF-α stimulation.**
(MP4)
