# Supplementary figures and images for: High-Throughput Analysis of NF-κB Dynamics in Single Cells Reveals Basal Nuclear Localization of NF-κB and Spontaneous Activation of Oscillations
Source: PLoS One. 2014 Mar 4;9(3):e90104. doi: 10.1371/journal.pone.0090104 (PMC3942427; doi:10.1371/journal.pone.0090104)

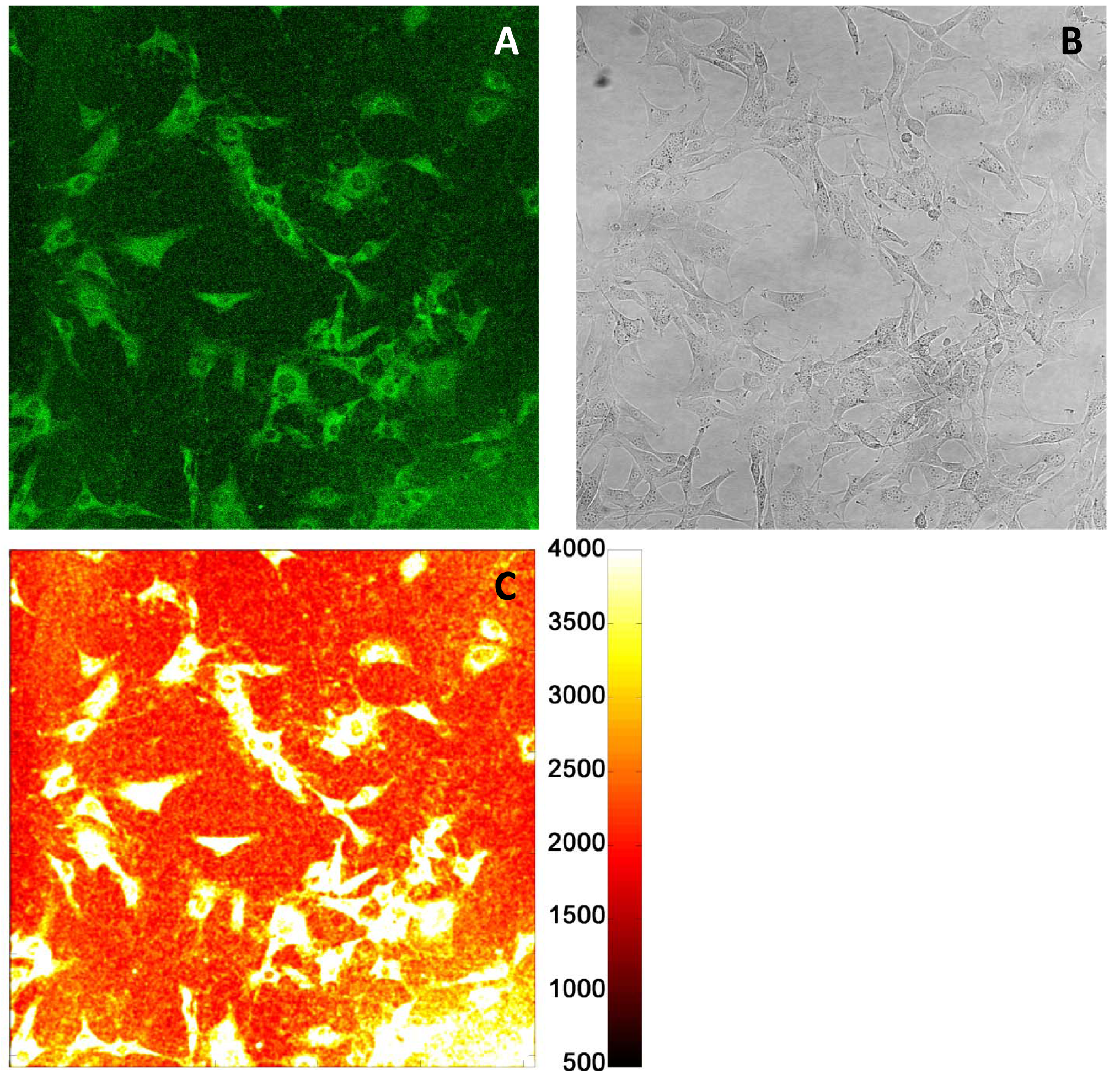

Supplement: Figure S1 — Cell autofluorescence has a negligible effect. NIH-3T3 and GFP-p65 MEFs are imaged in the GFP channel (A) to detect green fluorescence and in the transmission channel to detect all the cells in the field (B). In C is shown a different representation of A using a pseudo-color scale where high GFP intensities are extremely compressed (white for values from 4.000 to 20.000) while the signals in the lower range that approximate the background are represented on an extended scale and allow to appreciate minimal increases (orange). 3T3 cells are not observable in the GFP channel so we conclude that contribution of autofluorescence to our estimation of NT is negligible. (TIF) [file pone.0090104.s001.tif]

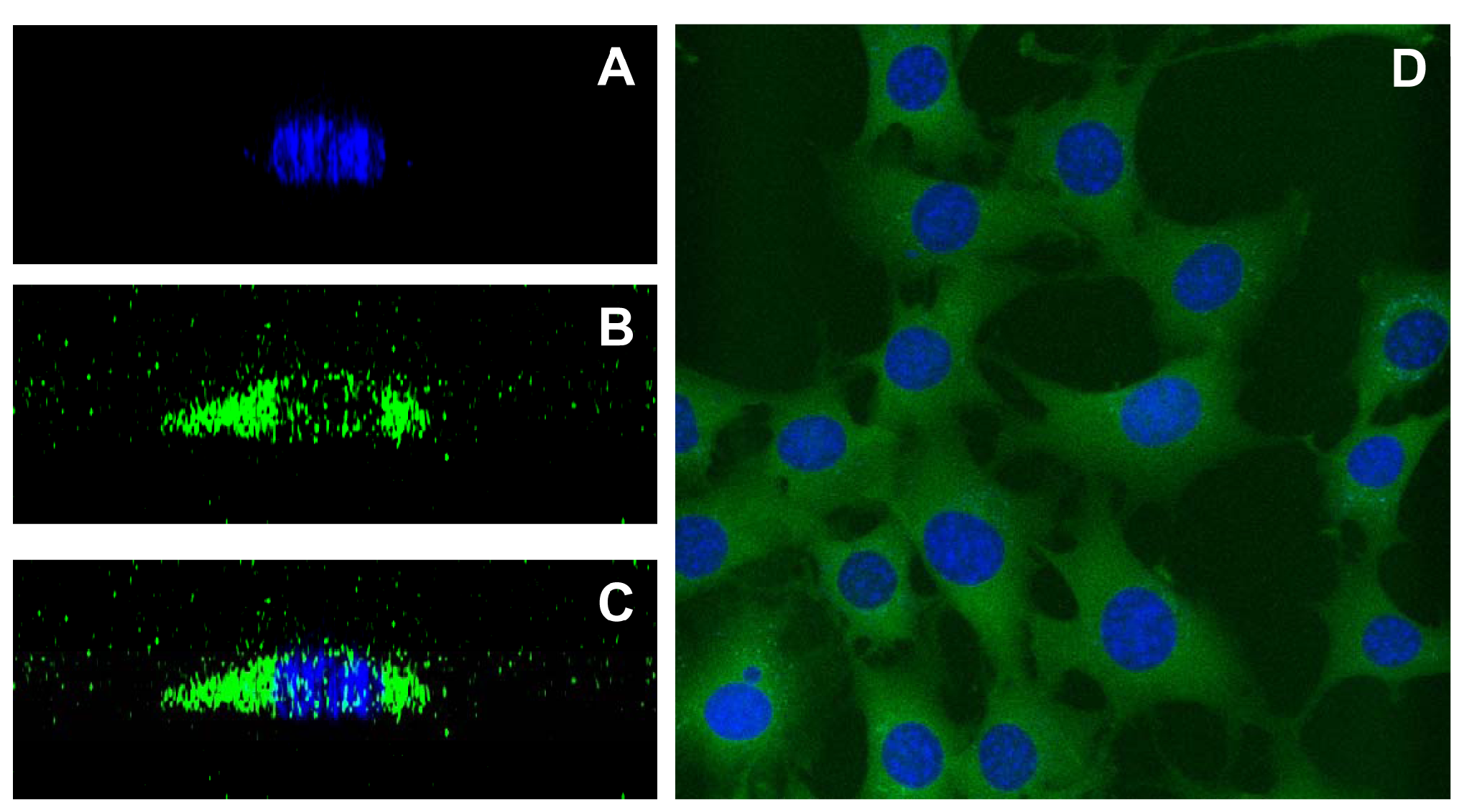

Supplement: Figure S2 — Additional data to support the existence of nonzero basal levels of nuclear NF-κB. Cell reconstruction using the z-stacks (see Materials and Methods) in (A) the HOE channel, (B) the GFP channel and (C) both, merged. We can see that the contribution of the cytoplasm above and below the nucleus is very small. Segmenting the “Sum slice” projection shown in (D) we obtain values of NT(0) = 0.15±0.06, compatible with our automated methods (mean and standard deviation computed for 30 cells). (TIF) [file pone.0090104.s002.tif]

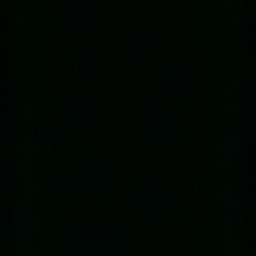

Supplement: Figure S3 — Example of a z-stack performed on unstimulated GFP-p65 MEFs. Z-stacks have been acquired with a 63x obj. and a z-width of 500 nm. Each cell has been segmented in the Hoe and the GFP channels to quantify the GFP fluorescence in nuclei and in the whole cells in each plane of the z-stack. By summing nuclear and cytoplasmic intensities from the whole stack for all the cells we get a value NT(0) = 0.11±0.04 (mean and standard deviation computed for 10 cells). Note that this segmentation is affected by inherent errors due to the imprecise identification of boundaries in planes with low fluorescence (top and the bottom of the cells; see also Figure S2). Moreover, z-stack analysis exposes the cells to possible phototoxic effects and cannot be applied for long time-lapses. The provided z-stack file is in tiff format and can be opened in ImageJ. Green and blue channels can be independently regulated to appreciate the contribution of each component. (TIF) [file pone.0090104.s003.tif]

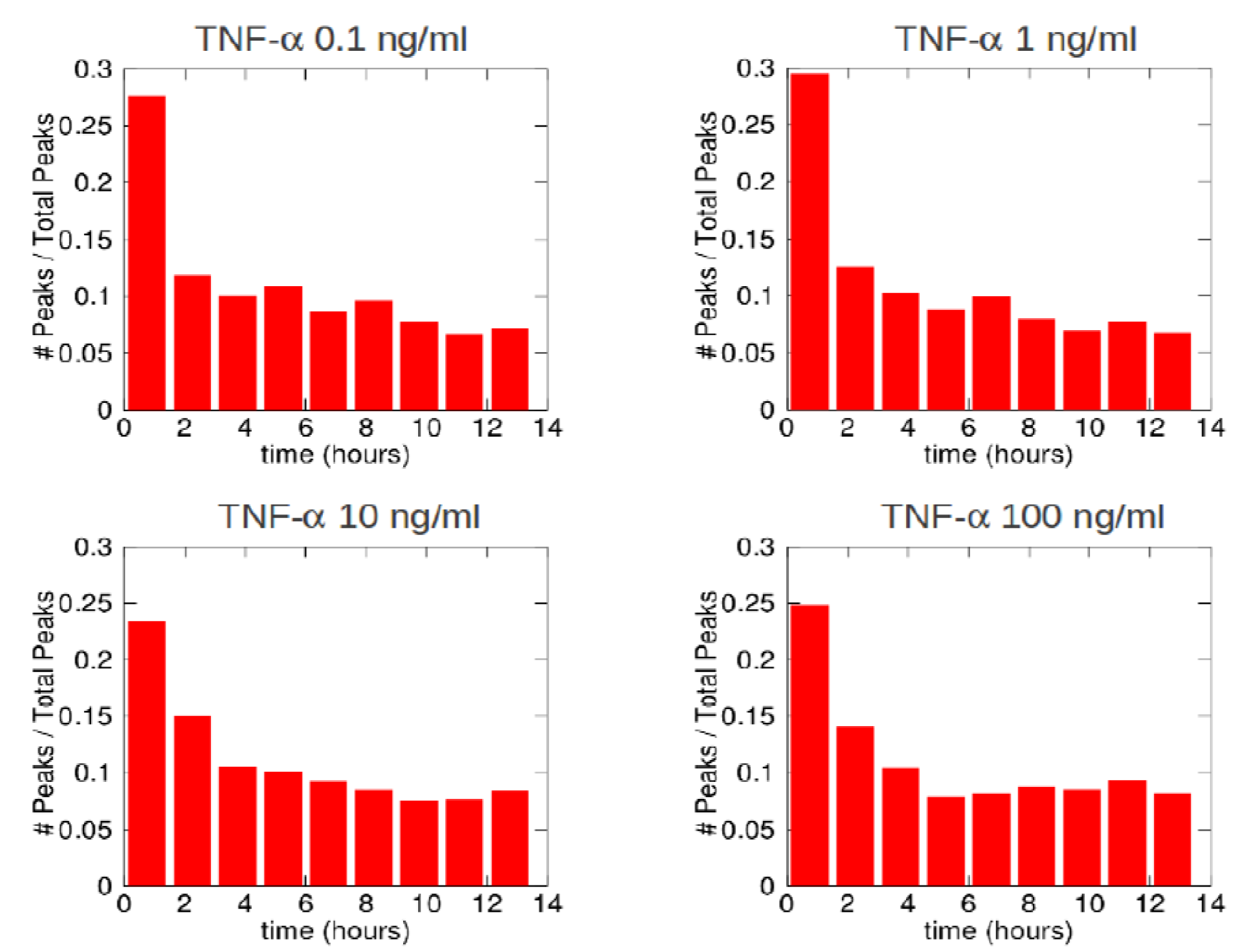

Supplement: Figure S4 — The response peak occurs in the first 2 hours after stimulation. Distribution in time of the significant peaks observed for cells using different stimulations (e.g. for 100 ng/ml TNF-α, 25% of peaks are in the first 2 hrs). When applying a test for uniformity of the timing, we always get p-values<0.001. (TIF) [file pone.0090104.s004.tif]

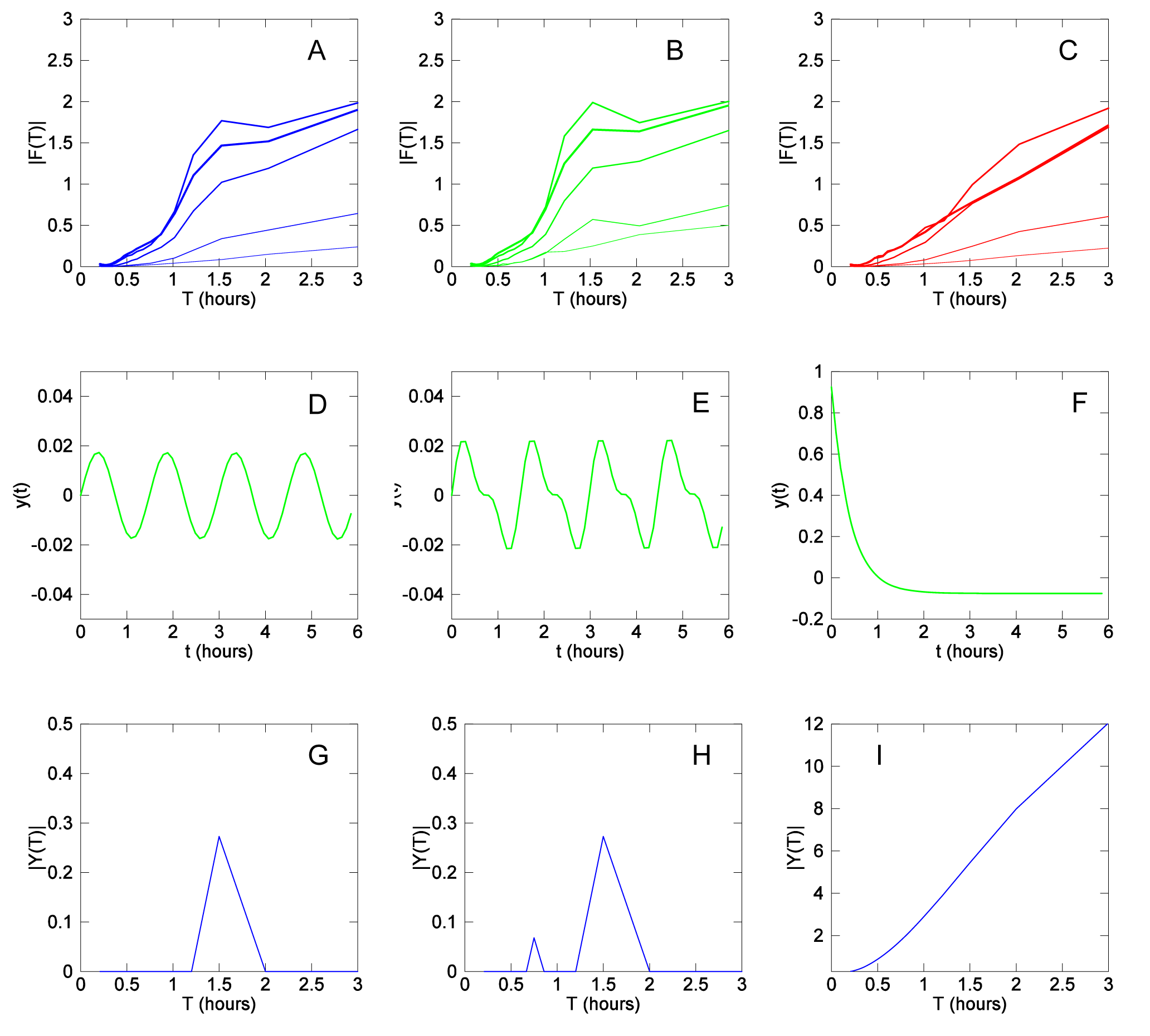

Supplement: Figure S5 — Spectral analysis of the time series confirms a T = 1.5 h periodicity in the dynamics. (A) Average periodograms for the time series obtained for the different doses used. In the periodograms we plot the amplitude of the Fourier transform for each period T. Average for (B) only oscillating cells and (C) non-oscillating cells for the doses of TNF-α considered. Thicker lines correspond to higher doses. Periods close to T = 1.5 hours are enhanced, and this enhancement is more evident for oscillating cells. The high period (low frequency) enhancement is due to the dampening observed for the average of the time series. (D) Sinusoidal time series (E) superposition of a high and low frequency sinusoidal time series and (F) series of an exponential decay. The time series have the same number of points that the series used for the periodograms. In (G), (H) and (I) we show their respective periodograms. The pure frequencies can be easily discerned. On the other hand, the high period (low frequency) enhancement observed in the periodogram of the exponential decay can be related with the one observed in the average periodograms of the time series of NT: which correspond to the one that would be observed for the damped average time series. (TIF) [file pone.0090104.s005.tif]

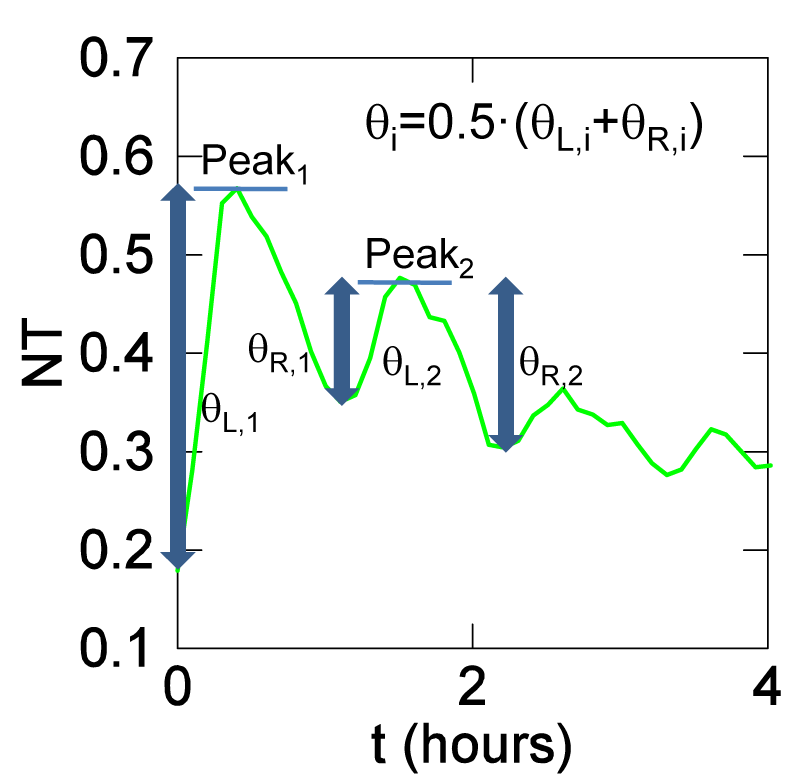

Supplement: Figure S6 — An example of a time series with high Peak2/Peak1 and lower. θ2/θ1. The ratio between the second and the first peak heights (Peak2/Peak1) is close to 0.8. The peak values of each peak are calculated as θi = 0.5·(θL,i+ θR,i), for the first peak θ1 it is close to 0.3 and for the second θ2 is close to 0.15, so the ratio between the peak values is close to 0.5. This situation is common in our time series and thus in general θ2/θ1 is smaller than Peak2/Peak1. (TIF) [file pone.0090104.s006.tif]

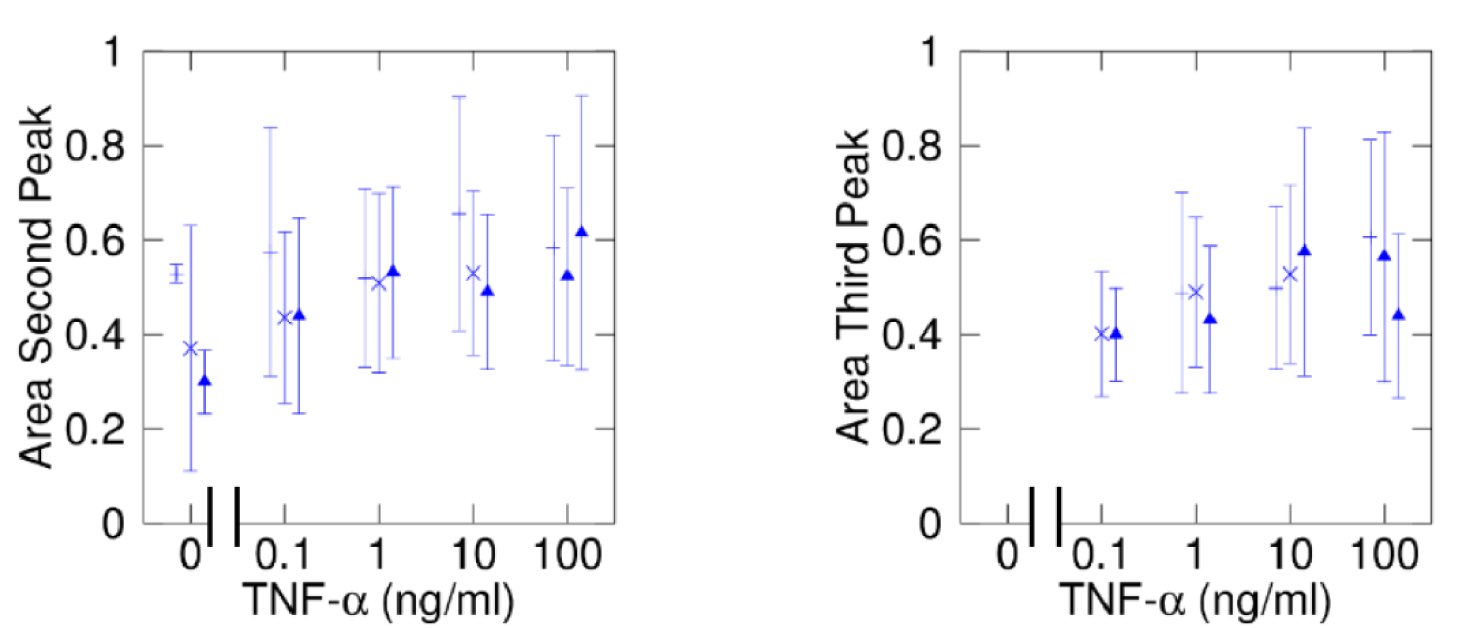

Supplement: Figure S7 — Area values from the second and third peaks. Area values under the second and the third peaks (when observed) are plotted for different TNF-αconcentrations. On average, the area values are remarkably constant. (TIF) [file pone.0090104.s007.tif]
