# Supplementary material for: High-Throughput Analysis of NF-κB Dynamics in Single Cells Reveals Basal Nuclear Localization of NF-κB and Spontaneous Activation of Oscillations
Source: PLoS One. 2014 Mar 4;9(3):e90104. doi: 10.1371/journal.pone.0090104 (PMC3942427; doi:10.1371/journal.pone.0090104)
Supplement: Table S1 — For each experiment performed we show the number of cells observed, the % selected, the average tracking time, the area (in pixels) of the nuclei and the NT value observed at time 0. We see that for all the experiments there is a nonzero basal level of nuclear NF-κB. Results are given as mean (standard deviation). (DOCX) [file pone.0090104.s008.docx]

**TABLE S1: Statistics describing the 15 experiment considered in this paper**

| **TNF (ng/ml)** | **Total cells** | **Selected** | **% Tracked** | **Avg tracking (h)** | **Area(0) (pixels)** | **Nuclear to Total Ratio (0)** |
| --- | --- | --- | --- | --- | --- | --- |
| 0 | 452 | 133 | 29.4 | 12.5 | 545 (98) | 0.14 (0.05) |
| 0 | 386 | 117 | 30.3 | 12 | 488 (96) | 0.17 (0.06) |
| 0 | 629 | 125 | 19.9 | 9.8 | 557(160) | 0.15 (0.05) |
| 0.1 | 307 | 98 | 31.9 | 11 | 523 (108) | 0.17 (0.05) |
| 0.1 | 634 | 137 | 21.6 | 9.9 | 503 (112) | 0.15 (0.05) |
| 0.1 | 404 | 106 | 26.2 | 10.1 | 512 (109) | 0.17 (0.08) |
| 1 | 416 | 165 | 39.7 | 11.2 | 528 (145) | 0.17 (0.05) |
| 1 | 604 | 180 | 29.8 | 10.3 | 517 (120) | 0.17 (0.05) |
| 1 | 335 | 142 | 42.4 | 11.8 | 540 (106) | 0.17 (0.05) |
| 10 | 530 | 176 | 33.2 | 10.6 | 532 (122) | 0.17 (0.06) |
| 10 | 639 | 194 | 30.4 | 10.6 | 500 (100) | 0.18 (0.06) |
| 10 | 442 | 94 | 21.3 | 8.8 | 489 (95) | 0.15 (0.05) |
| 100 | 410 | 87 | 21.2 | 8.6 | 527 (104) | 0.15 (0.05) |
| 100 | 444 | 79 | 17.8 | 7 | 485 (105) | 0.17 (0.06) |
| 100 | 255 | 106 | 41.6 | 8.4 | 518 (97) | 0.20 (0.09) |

T**able S1.**

We show in Table 1 the total cells observed, the % selected, the average tracking time, the area (in pixels) of the nuclei and the NT value observed at time 0. We see that for all the experiments there is a nonzero basal level of nuclear NF-kB. Results are given as mean (standard deviation).
