# Supplementary material for: High-Throughput Analysis of NF-κB Dynamics in Single Cells Reveals Basal Nuclear Localization of NF-κB and Spontaneous Activation of Oscillations
Source: PLoS One. 2014 Mar 4;9(3):e90104. doi: 10.1371/journal.pone.0090104 (PMC3942427; doi:10.1371/journal.pone.0090104)
Supplement: Document S1 — Description of the software used for the single-cell quantification of NF-κB dynamics. (DOC) [file pone.0090104.s009.doc]

**Supplementary Document 1: Description of the software.**

Our time-lapse data consist of images of our cells captured for a number of frames *Nframes* that we can label with the natural index *t* ranging from *1* to *Nframes*.

We provide here a more detailed description of the operations performed by our software in each frame.

*I. Conversion of images into matrices.*

Convert the *M x N* pixels images from the HOE channel (the image of the stained nuclei) and the GFP channel (the image of GFP-p65) in *M x N* matrices (we work here with matrices of size *M=N=1024*), that we call *matrixHOE* and *matrixGFP* respectively.

*II. Calculation of an approximate threshold for the nuclei.*

- After applying a Gaussian filter to *matrixHOE*, divide it in tiles of size *L* and cluster them according to their intensities using a K-means algorithm.
- Determine an approximate threshold as the mean of the average intensity of the centroids of the *k<K* clusters with a higher mean (*L=32*, *K=2* and *k=1* worked properly for our calculations).

*III*. *Thresholding of nuclei.*

- - Obtain a MxN matrix of 0s and 1s by thresholding the *matrixHOE*: pixels of *matrixHOE* with values bigger than the approximate threshold calculated in step *II* are marked by a 1. Eliminate isolated pixels of 1s (those surrounded by 0s)
  - Call the resulting matrix of 0s and 1s *matrixthreshold* (it is plotted in **Figure 2C** of the manuscript).

*IV. Approximate segmentation of the nuclei.*

- A **cluster** of 1s in a matrix of 0s and 1s is defined as a set of nonzero elements in a matrix such that for any two elements of the cluster *x* and *y*, it is possible to go from *x* to *y* by making displacements of at most one row and one column each step without passing through a position with value 0.
- Identify the clusters of 1s in *matrixthreshold*, and label them with an integer number from 1 to *Nnuc*.
- Discard those whose areas (numbers of pixels) are not adequate (adjustable by the user) or touch the border of the image.
- Store the area of each cluster found. Store the **position** of each cluster as a four-component vector *[min_row, max_row, min_colum, max_colum]* containing the values of the rows and the columns between which the cluster is found. The dimension of the object in the rows and in the columns direction will be *max_row-min_row* and *max_row - min_row,* respectively*.* Store all the positions in a *Nnuc x 4* matrix so that the *m*-th row gives the position of nucleus with label *m*.
- Generate a matrix of integers, *matrixnew*, a matrix such that the number written in each position is the label of the cluster to which it belongs (zero if it belongs to no cluster). A color plot of this matrix is shown in the figure below.

*
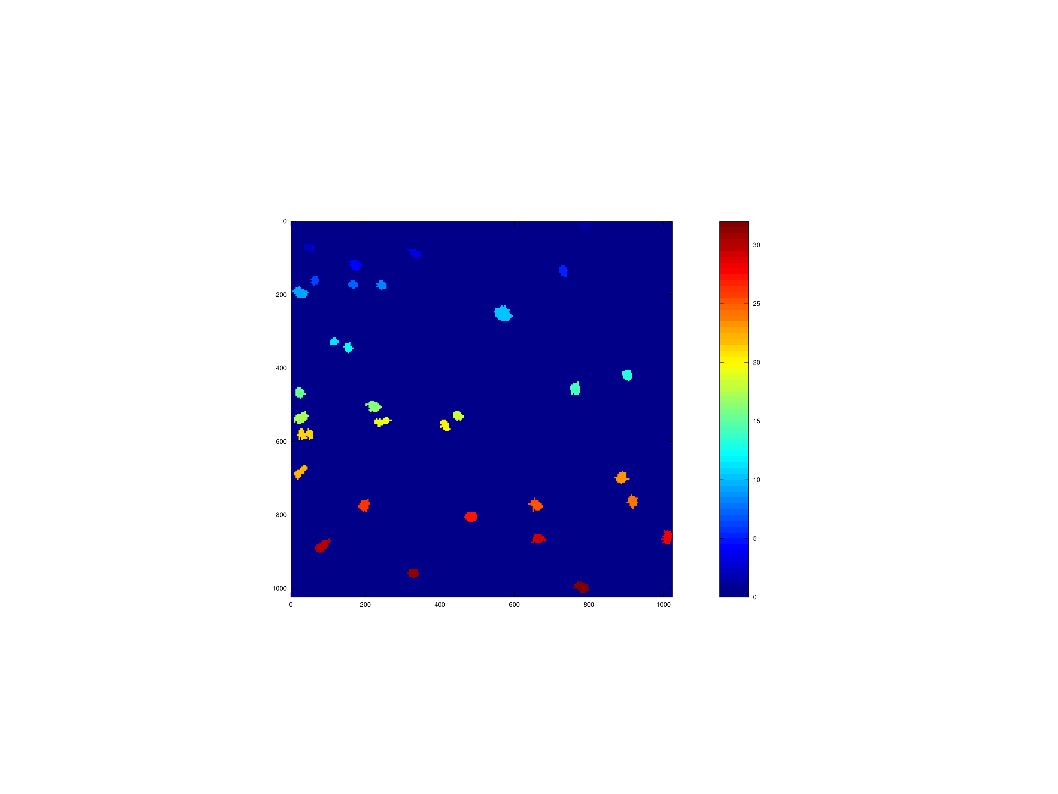
*

*FIGUREMATRIXNEW.EPS*

*IV.bis: In the first frame, prepare for data storage.*

- keep the list of elements *listnuclei=[1,....,Nnuc]* because these will be the nuclei that have to be tracked in subsequent frames. This list is updated each frame. When a cell (a nucleus) is not tracked anymore, its integer label is extracted from the list.
- Three dimensional matrices are objects with three indexes *i,j,k*: *i* indicates the **row**, *j* the **column** and *k* the **slice**. Each slice of this matrix, then, can be seen as a “normal” two-dimensional matrix.
- Create a matrix of size *M x N x Nframes*, where *Nframes* is the number of frames. As we will show below, in this three dimensional matrix where we store the results of our detection of nucleus and cytoplasms: call it *matrixresults3D*.
- Create a matrix of size *4 x Nnuc x Nframes*, called *matrixposnuc3D.* In the *n*-th row of the slice *t* we will store the position of the nucleus *n* at time *t*. We will use a matrix of the same size, *matrixposcyto3D* to store the positions of the cytoplasms around each nuclei for each tracked cell (when in a given frame the nucleus or the cytoplasm is not tracked anymore we will just write [-1 -1 -1 -1]).
- Create matrices of size Nnuc x Nframes so the element *m,*t will be the area, the nuclear intensity (without background), the cytoplasmatic intensity (without background), respectively, of the cell n in the frame *t*.

*V. Precise segmentation of the nuclei.*

- If we are not in the first frame, relate the nuclei detected in this frame with the nuclei of *listnuclei* considering their positions in the previous frame. This is done by looking for the closest nucleus in the previous frame (the positions will be in the slice of *matrixpos3D* corresponding to the previous frame). If the correspondence for some nucleus cannot be established, delete it from the list of object tracked, *listnuclei*.
- Once the correspondence has been established, go to the positions of each nucleus in *listnuclei*, average the value of *matrixHOE* in a small window located in the approximate position of each nucleus (size adjustable by the user depending on the homogeneity of the nucleus). This value is used for thresholding and segmentation (we check for the bigger cluster in a small window centered in the nucleus position).
- Check the area of the new object by counting the number of pixels. If the area of the object changed too much (tolerance adjustable by the user, in our case we use a tolerance of 30% of relative change in size) then take it away from *listnuclei*.
- Generate a *MxN* matrix called *matrixgoodObject* such that the number written in each position is the label of the cluster of the precisely segmented nucleus to which that position belongs (zero if it belongs to no cluster; see **Figure 2E** of the manuscript).
- Generate a matrix such that in row *m* we will store the position of each nucleus detected. If the nucleus is found to touch the border of the images, remove it from *listnuclei*.

*VI. Calculation of the background.*

- Divide *matrixGFP* in tiles of side *L* (we used *L=32*), discard the tiles that overlap with windows (of size adjustable by the user) centered in the positions of the nuclei approximately detected (those that are displayed in our color plot of *matrixnew*). (In our case, we take windows of dimensions *3I* and *3J* centered in the center of each nuclei, where *I* and *J* are the dimensions of the object in the rows and arrows direction, respectively).
- In what follows, we proceed as in Schwarzfischer *et al.* (2011) (Ref. [18] of the manuscript) for the background calculation: we cluster the tiles using a Density-Based Scan Algorithm with Noise (DBSCAN) based in the relative distances of vectors built using the following statistical parameters: the normalized standard deviation, skewness, maximum to minimum ratio, kurtosis of the distribution of pixel values in each tile.
- Take the cluster of tiles with smaller mean: these tiles are those belonging to the background. Reconstruct the background of all the image by interpolating between tiles to cover the holes. We get thus *matrixBG*.
- There might be zones of *matrixBG* that cannot be filled by interpolation. In that case the value of the background is set to zero.

*VII. Determination of the cytoplasm and calculation of NT.*

- For each nucleus in *listnuclei* having dimensions *m x n*, take a window centered on it of size *3m x 3n* (it will be formed by 9 tiles of size *mxn*, the center one enclosing the nucleus). Take the average pixel value greater than the background in each tile except for the center one (the one containing the nucleus).
- This value will be the threshold used to estimate the cytoplasm of each nucleus: each time we look for the cytoplasm of a cell, we create a matrix of zeros *matrixapproxcyto* in such a way that when for a given position in matrixGFP the value is above the threshold, we write a 1 in the same position of the corresponding position of *matrixapproxcyto*. This thresholding is only done in a window of size *L* (*L=200* in our calculations) around the nucleus of interest. The clusters of 1s thus generated are detected. The cytoplasm will be the cluster(s) overlapping with the nucleus, if any. If none is found, we take the label of the nucleus from *listnuclei*.
- The cell is discarded and thus its label is removed from *listnuclei* if the cytoplasm is found to overlap with some of the clusters detected in *matrixnew* (this occurs typically when two cells touch), or if the cytoplasm is found to touch the border of the image, or if the *mabrixBG* is zero (meaning that we were not able to estimate the background in a given point) for some of the positions of the windows around the nucleus considered before.
- Once the nucleus and the cytoplasm are known, provided that we know *B(p,t)* and *I(p,t)*, we can use equation (3) of the manuscript to estimate NT.
- The matrix *matrixcyto* is a matrix whose nonzero elements correspond to the cytoplasms around each tracked nucleus. The number written in those positions is *Nnuc+n*, where n is the label of the nucleus considered.
- The matrix *matrixgoodObjectdef* is exactly the same as *matrixobject* but setting to zero the clusters corresponding to nucleus for which the cytoplasm could not be detected.
- Generate a matrix *matrixpositionnucdef* with the positions of the nucleus for which we have detected the cytoplasm.
- Similarly, generate a matrix *matrixpositioncyto* with the positions of the cytoplasms detected properly.

*VIII. Calculation of NT and Information storage*

- We create a matrix called *matrixsummaryresults*. It is a matrix of zeros in which the only nonzero elements are: the nonzeroelements of *matrixnew*, multiplied by -1, those of *matrixcyto* and of *matrixgoodObjectdef.* with their same values. The resulting matrix, plotted below, will be stored in the slice of *matrixresults3d* corresponding to the current frame. This matrix clearly summarizes all the information obtained for the tracking and the segmentation procedures.


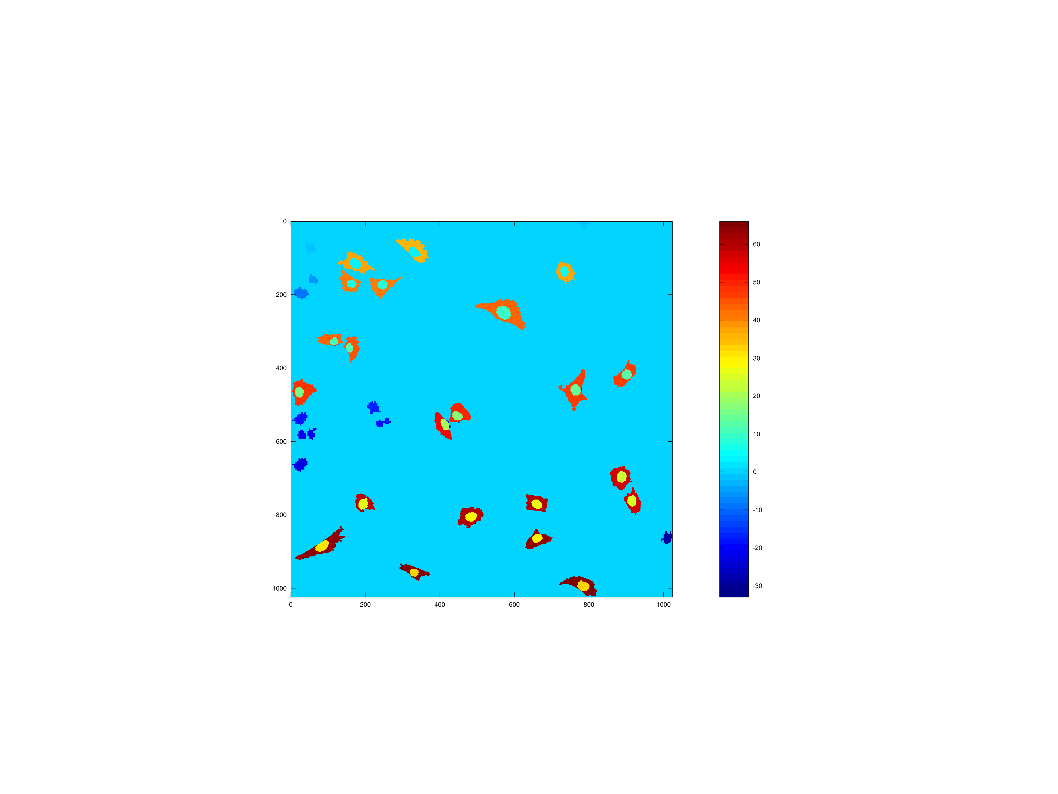


- We store the matrices of the positions of the cytoplasm and the nucleus, *matrixpositioncyto* and *matrixpositionnucdef* , in the corresponding slice of *matrixposnuc3D* and  *matrixposcyto3D,* respectively*.*
- Store in the corresponding matrices the nuclear intensity, the cytoplasmic intensity, the NT value, the area of the nucleus and the area of the cytoplasm.

This process is repeated for each frame until we have reached the last frame or, alternatively, when there are no elements in *listnuclei*.
