# Supplementary material for: High-Throughput Analysis of NF-κB Dynamics in Single Cells Reveals Basal Nuclear Localization of NF-κB and Spontaneous Activation of Oscillations
Source: PLoS One. 2014 Mar 4;9(3):e90104. doi: 10.1371/journal.pone.0090104 (PMC3942427; doi:10.1371/journal.pone.0090104)
Supplement: Document S2 — Relation between different quantifiers of NF-κB dynamics used in the literature. (DOC) [file pone.0090104.s010.doc]

**Supplementary Document 2. Relation between different quantifiers of NF-kB dynamics used in the literature.**

In different works where the nuclear to cytoplasmatic ratio of the protein is calculated, that can be defined as:

$NC(t)=\frac{\sum_{p \in nucleus} P(p,t)}{\sum_{q \in cytoplasm} P(q,t)}$ (S2.1)

It is quite easy to show that the nuclear to cytoplasmatic ratio of the protein *NC(t)* and the nuclear to total ratio that we calculate *NT(t)* can be related through the expression:

$NC(t)=\frac{NT(t)}{1-NT(t)}$ (S2.2)

Sometimes it is the nuclear to cytoplasmatic ratio of the *intensities* what is calculated. This is presumably calculated as

$NCI(t)=\frac{\frac{1}{A_{nucleus}}\sum_{p \in nucleus} P(p,t)}{\frac{1}{A_{cytoplasm}}\sum_{q \in cytoplasm} P(q,t)}$ (S2.3)

It is easy to see that the following equality follows

$NCI(t)=\frac{A_{cytoplasm}}{A_{nucleus}}NC(t)$ (S2.4)

This measure would be as robust to experimental distortions as *NT*. In **Figure 3** we plot the values of *NC*, *NCI* that would be obtained for different values *NT*. In particular, a *NT* value in the range 0.5 - 0.75 (that we obtain for stimulation with TNF-α) correspond to values 1-3 of *NC* and 2-6 of *NCI* (in our estimation of *NCI* we assume that in the images the area –number of pixels- of the cytoplasm is twice that of the nucleus). This over-amplification of high values has made us think that *NT* is a preferable quantifier of the dynamics of NF-kB.
