# Supplementary material for: High-Throughput Analysis of NF-κB Dynamics in Single Cells Reveals Basal Nuclear Localization of NF-κB and Spontaneous Activation of Oscillations
Source: PLoS One. 2014 Mar 4;9(3):e90104. doi: 10.1371/journal.pone.0090104 (PMC3942427; doi:10.1371/journal.pone.0090104)
Supplement: Document S3 — Pseudocode for the detection of significant peaks. (DOC) [file pone.0090104.s011.doc]

**Supplementary Document 3. Pseudocode for the detection of significant peaks**

The aim of this pseudocode is to find sequences of local minimum, local maximum and local minimum {*min_L_, max, min_R_*} (each of them specified by a position and a value in the time series) that determine a significant peak above a threshold θ. 

**1**. Find the local maxima and the local minima of the series *{...min_n_,max_n_,min_n+1_,max_n+1_...}*. In the first step, the first point of the time series is considered a minimum if the time series is increasing right after it. Similarly, it will be considered a maximum if it is decreasing right after it. Similarly, the last point of the time series is considered as a minimum if the time series is decreasing right before it, and a maximum if it is increasing right before it.

**2.** Start from *n’*, *n’=1* the first time, take *min_n’_*  and store its value as *min_L_*. Go for values of the list with *n>n’*.

**2.1** If a *max_n_* is found such that *max_n_ - min_L_* *>* θ then store the value as *max*, so we have *{min_L_, max}* . Set *n’’=n* and continue searching for values of *n>n’’*.

**2.1.1** If a minimum *min_n_* such that *max-min_n_ >* θ is found, save the value as *min_R_*. Store the significant peak {*min_L_, max, min_R_*}. Start from *n’=n* the search of a new peak.

**2.1.2** If a maximum *max_n_* such that *max_n_* >*max* is found, use it to replace the value of max {*min_L_, max*} and go back to 2.1 setting n’’=n.

**2.2** If a min*_n_* is found such that min*_n_* <min, go back to 2 setting *n’=n*. After all the elements of the list have been examined, stop if the length of the list has not changed or if the list is empty (this will happen when no significant peaks are observed).

A list of the observed significant peaks of the time series, stored each as {*min_L_, max,min_R_*} is thus retrieved.
